# Electroanalytical point-of-care detection of gold standard and emerging cardiac biomarkers for stratification and monitoring in intensive care medicine - a review

**DOI:** 10.1007/s00604-022-05186-9

**Published:** 2022-03-12

**Authors:** Robert D. Crapnell, Nina C. Dempsey, Evelyn Sigley, Ascanio Tridente, Craig E. Banks

**Affiliations:** 1 Faculty of Science and Engineering, Manchester Metropolitan University, Chester Street, Manchester, M1 5GD UK; 2 Intensive Care Unit, Whiston Hospital, St Helens and Knowsley Teaching Hospitals NHS Trust, Warrington Road, Prescot, L35 5DR UK

**Keywords:** Biosensor, Nanomaterial, Electrochemistry, Electroanalysis, Cardiac biomarkers, Critically ill, Intensive care

## Abstract

Determination of specific cardiac biomarkers (CBs) during the diagnosis and management of adverse cardiovascular events such as acute myocardial infarction (AMI) has become commonplace in emergency department (ED), cardiology and many other ward settings. Cardiac troponins (cTnT and cTnI) and natriuretic peptides (BNP and NT-pro-BNP) are the preferred biomarkers in clinical practice for the diagnostic workup of AMI, acute coronary syndrome (ACS) and other types of myocardial ischaemia and heart failure (HF), while the roles and possible clinical applications of several other potential biomarkers continue to be evaluated and are the subject of several comprehensive reviews. The requirement for rapid, repeated testing of a small number of CBs in ED and cardiology patients has led to the development of point-of-care (PoC) technology to circumvent the need for remote and lengthy testing procedures in the hospital pathology laboratories. Electroanalytical sensing platforms have the potential to meet these requirements. This review aims firstly to reflect on the potential benefits of rapid CB testing in critically ill patients, a very distinct cohort of patients with deranged baseline levels of CBs. We summarise their source and clinical relevance and are the first to report the required analytical ranges for such technology to be of value in this patient cohort. Secondly, we review the current electrochemical approaches, including its sub-variants such as photoelectrochemical and electrochemiluminescence, for the determination  of important CBs highlighting the various strategies used, namely the use of micro- and nanomaterials, to maximise the sensitivities and selectivities of such approaches. Finally, we consider the challenges that must be overcome to allow for the commercialisation of this technology and transition into intensive care medicine.

**Graphical abstract:**

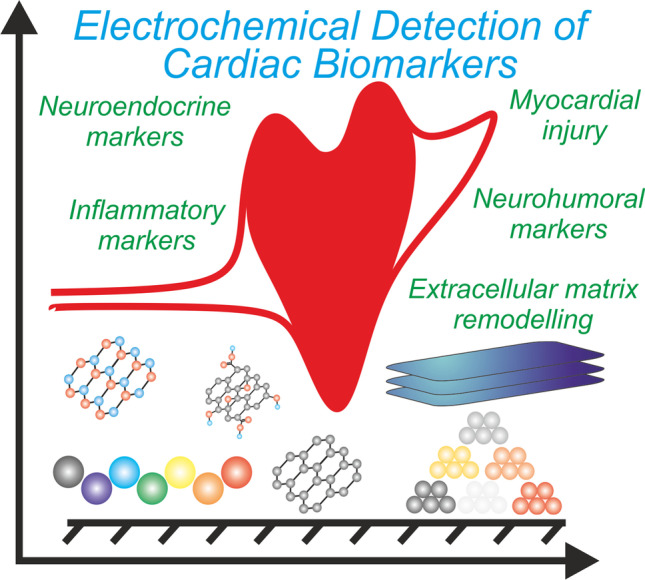

## Importance of rapid testing for cardiac markers in critically ill patients

Cardiovascular dysfunction is a frequent complication of critical illness. Approximately 30% of patients admitted to the intensive care unit (ICU) have underlying cardiac diseases, and approximately 50% of this group are admitted to the ICU with cardiac problems as the primary cause [1, 2]. This includes conditions such as acute myocardial infarction (AMI), heart failure (HF) and cardiogenic shock. However, cardiac complications can arise in ICU patients who have been admitted due to other critical illnesses such as sepsis [3], severe burns [4] and brain trauma [5]. ICU patients are exposed to high levels of non-cardiac stress, which in turn, increases myocardial oxygen consumption. In some patients, the myocardial oxygen supply may be reduced by hypotension, tachycardia, hypoxemia and anaemia. The heart is one of the most frequent organs to fail in critically ill patients [6, 7], and this can have several profound implications for a patient’s prognosis [8, 9]. As such, accurate assessment and monitoring of cardiac function in the ICU is vital to patient care.

Identifying cardiac dysfunction in critically ill patients, however, can be difficult. Co-morbidities and other confounding factors, along with the non-specificity of clinical symptoms, complicate diagnosis. Furthermore, the broad aetiologies behind cardiac dysfunction, are echoed by a wide range of cardiac pathophysiologies, and yet, prompt, appropriate stratification and treatment is crucial to patient outcome, since the acute nature of the dysfunction in many cases, can result in rapid patient deterioration and ultimately, death. Repeated monitoring of cardiac function over time is also vital and comprises assessment of the initial haemodynamic state, and ongoing evaluation of any change in this state that can indicate patient deterioration. Furthermore, assessment of the heart in response to administrated therapies is important in critically ill patients. Ideally, testing for cardiac dysfunction on the ICU should be performed using techniques that are rapid and can be performed repeatedly, with ease.

Cardiac biomarkers (CBs) are produced as a result of a pathological processes in the cardiovascular system. Several are now well-established and routinely used to aid diagnosis of a cardiac event, particularly within the emergency medicine setting, and to identify the progression of cardiovascular diseases [10–15]. Indeed, biomarkers of cardiac injury have been used to aid the diagnosis of AMI for over half a century, with aspartate transaminase (AST) being the first CB to be used in clinical practice [16]. However, the lack of specificity of AST for myocardial injury quickly saw it superseded by more clinically relevant CBs which could be used to identify AMI in an emergency room setting, and technologies for their rapid and accurate detection have been sought.

Although testing for certain CBs in emergency and cardiology settings is relatively commonplace, CB testing on the ICU is performed far less frequently. However, CB testing in critical illness has gained significant interest, with the hopes of providing useful information to supplement that provided by more conventional cardiac assessment methods. Indeed, diagnoses and patient stratification based on more traditional methods such as echocardiography are not always sufficient to inform the most appropriate treatment or management strategy. For example, while an enlarged right ventricle signifies pressure or volume overloading, imaging cannot aid in determining the aetiology. Similarly, ECG has been reported to have a low sensitivity for identification of AMI in the critically ill population. CBs, on the other hand aid the diagnostic process by providing information on the nature of the cardiac damage, for example suggesting myocardial stretch, inflammation or cardiomyocyte necrosis.

CBs are also important for prognostication and risk stratification in critically ill patients, with specific CBs measured early in the patient’s ICU stay repeatedly being shown to predict outcome in specific ICU subgroups. Indeed, Cardiac Troponin I (cTnI) strongly predicts mortality and/or length of hospital stay such as in the case of trauma [17], sepsis [18], pneumonia [19] and COVID-19, [20] although, cTnI should be considered more as a specific marker / “gold standard” for the diagnosis of AMI [21]. Likewise, other CBs such as Cardiac Troponin T (cTnT) [22, 23], B-type natriuretic peptide (BNP) [24], N-terminal (NT)-pro hormone BNP (NT-proBNP) [25, 26], soluble suppression of tumourigenicity 2 (sST2) [27, 28] and Heart-type Fatty Acid-Binding Protein (H-FABP) [29], have all been shown to demonstrate some degree of prognostic value in critically ill subgroups. This could be extremely useful for daily practice on the ICU since early measurement of specific CBs may help clinicians to identify early risk of deterioration and may allow for optimisation of ICU resources. Rapid, cost-effective identification of CBs on the ICU therefore holds great potential, since CB testing in this setting is under-utilised. Although point-of-care (PoC) systems for rapid analysis of Troponins and some natriuretic peptides have been adopted in some settings for ruling-out AMI in patients presenting with acute chest pain, some of these technologies are costly, and all other CBs must be analysed by clinical chemistry analysers or plate-based immunoassay in the hospital pathology department.

When considering the development of technology for the rapid detection of CBs, it is vital that the most appropriate CBs are targeted, and this is particularly relevant when considering ICU patient groups; there are a vast number of CBs encompassing enzymes, hormones, and proteins, each with their own set of key attributes and supporting literature; see Table 1. Some of these are more applicable for patient diagnosis, while others hold greater prognostic significance. It is also extremely important to consider that CBs are generally elevated in the critically ill patient population overall (see Table 1) and so require the derivation of separate reference ranges for this distinct cohort. Technologies adopted in the ICU will therefore need to cover a much broader analytical range, than those used to assess CBs in the emergency department for rule out of AMI/HF. Table 1 summarises the evidence for the clinical utility of CBs in ICU patients and provides an indication of the concentrations of each reported in this patient group; from this critically useful table, we overview the electroanalytical approaches to their determination, providing an up-to-date overview.

**Table 1:** Cardiac biomarkers, their source, clinical relevance, and analytical ranges

	Biomarker		Source	Examples of clinical relevance	Remarks	Analytical ranges
**Markers of myocardial injury**	Cardiac Troponins	cTnT	Cardiomyocytes & skeletal muscle (although the isoforms from both, differ)	• Troponins are the most widely used biomarkers in clinical care • Although cTnT and cTnI have very high sensitivity for AMI in the ED [10] [11], and are included in NICE guidance for early rule out of NSTEMI [30], in ICU patients the incidence of raised cTnT and cTnI is high (45%), and 70% of these patients do not have flow limiting coronary artery disease • cTnI strongly predicts mortality and/or length of hospital stay in trauma [17], sepsis [18], pneumonia [19] and also COVID-19 patients [31], and can strengthen existing prognostication systems such as APACHE II [32] • The presence of cTn in the blood of critically ill patients may identify sub-cohorts who can benefit from different treatment approaches [33]	• Not seen until 6–8 h post myocardial injury, with concentrations peaking at around 12–24 h [34] • Levels can remain elevated for 7–10 days post original myocardial insult, and so this late clearance makes it difficult to identify a recurrent cardiac event [34]	• Normal values reported as 99th centile: 14 ngL^−1^ [35] • Studies assessing the prognostic value of cTnT in ICU patients have frequently used > 10ngL^−1^ as the cut-off [36]
		cTnI	Cardiomyocytes			• Normal values reported as 99th centile: 34 ngL^−1^ in men, 16 ngL^−1^ in women [35] • Studies assessing the prognostic value of cTnI in ICU patients have frequently used > 100ngL^−1^ as the cut-off [36]
	H-FABP		Cardiomyocytes, skeletal muscle, brain and kidney	• Previously shown to be effective for diagnosis of ACS and AMI [11, 37, 38] • Proven utility for prediction of PE in ICU patients [39, 40] • Demonstrates prognostic significance in ED patients [29] and also sepsis patients on the ICU [41] • Elevated in critically ill patients, but can predict the development of adverse cardiac events in trauma patients when measured upon admission to the ICU [42]	• Present in circulation much earlier following cardiac injury when compared with Troponins [43], being evident at just 30 min post-injury and peaking at 6–8 h • Rapid clearance, with H-FABP levels returning to baseline approximately 24 h post-cardiac event [44] • However, recent large studies have shown that H-FABP does not improve hs-troponin diagnostic accuracy and that its incremental value over hs-troponin has uncertain clinical significance [45, 46]	• Typically, the normal range is considered as: < 5 ng mL^−1^. However, at least two different thresholds have been defined as the 99^th^ percentile of a healthy population, and several cut-off values for H-FABP positivity have been used [47] • The lower the positivity threshold, the better the H-FABP performance, thus, optimal sensitivity has been mostly reported at a cut-off of 4 ng mL^−1^ [47]. • It has recently been shown that H-FABP is significantly higher in sepsis patients (median 26.6 (range 9.3 to 79.0) ng mL^−1^) than in those with compensated HF (median 6.6 (range 4.6 to 9.7) ng/ml), highlighting the wide analytic range required for critically ill patients [48]
	CK-MB		Cardiomyocytes, skeletal muscle, brain and kidney	• Introduced in 1965 as a biochemical marker for myocardial damage—one of the oldest markers in this field [49]. • Although it has high specificity for diagnosis of AMI, it has low specificity, and hence is not used in isolation • Repeatedly been shown to demonstrate strong prognostic significance in COVID-19 patients [50]	• Released within 12 h after symptom onset of AMI, peaks in serum at 24–36 h, and returns to normal in 48–72 h – not suitable for early diagnostics in the setting of acute chest pain • CK-MB may appear raised in other conditions such as rhabdomyloysis or stroke. For this reason, CK-MB is measured in conjunction with Total CK and other cardiac markers such as Troponin T and/or Myoglobin to produce a clearer clinical picture	• Normal range: Male: 0–5.0 ng/mL, Female: 0–2.9 ng mL^−1^ [51]. • Concentrations of > 60 ng mL^−1^ have been reported in ICU patients, particularly those with hypovolaemic shock
	Myoglobin			• Myoglobin kinetics (detecting a change of 40 ng/ml) within the first hour of acute chest pain is reported to have a 91% sensitivity and up to a 99% negative-predictive value for AMI [52] • It should be noted that myoglobin testing has largely been discontinued in clinical laboratories since cTnT or cTnI assays have increased in sensitivity and is generally viewed as an outdated diagnostic marker • Myoglobin has been shown to possess prognostic value in sepsis [53] and COVID-19 patients, and may be superior to cTn in that respect [54, 55]	• Elevated levels are normally detectable between 2 and 6 h after MI peaking within 5–18 h. It is generally detectable before CK-MB and cTn [56]	• Normal range: 25–90 ng mL^−1^ [56] • Ideally, an assay for myoglobin should have a sensitivity of < 5 ng mL^−1^ and a dynamic range of at least 500 ng mL^−1^ [56]
**Neuroendocrine markers and indicators of myocardial stretch**	BNP		Cardiomyocytes of ventricle	**•** Signifies ventricular myocardial stretch and is hence useful in diagnosis of HF • Increased BNP level is a strong predictor for cardiac dysfunction in ICU patients [2] • Can aid in the diagnosis of cardiac dysfunction in ICU patients, but cannot replace echocardiography—merely indicates the presence of a ‘cardiorenal distress’ and should prompt further investigation [57] • Also shown to be useful for prognostication in sepsis and COVID-19 [24, 58]	• In response to myocardial wall stretch, pre-proBNP is synthesised and processed to proBNP; which is further processed to the biologically inactive NT-proBNP fragment and the biologically active BNP fragment • Circulating BNP levels are similar to NT-proBNP in normal individuals but are significantly less elevated by left ventricular dysfunction than NT-proBNP [59].	• Mean (SD) BNP in healthy controls is reported as 56.87 ng/L (22.76ngL^−1^) [24] • BNP < 100 ngL^−1^, CHF unlikely [59]. • BNP 100–500 ngL^−1^—equivocal range [59]. • BNP > 500 ngL^−1^ consistent with the diagnosis of CHF [59]. • Values of ≈500ngL^−1^ reported in critically ill patients without diagnosis of cardiac complication [24, 58]
	NT-proBNP		Cardiomyocytes of the ventricles	• NICE guidance recommends NT-pro-BNP for early rule out of NSTEMI [60] • NT-proBNP is considered the gold standard biomarker in HF diagnosis and management [61] and is recommended as part of diagnostic workup in the European Society of Cardiology [ESC] Clinical Practice Guidelines and the American AHA/ACC/HFSA Guidelines • RV pressure overload due to acute PE is associated with increased myocardial stretch, and hence NT-proBNP. Thus, the plasma levels of NT-proBNP reflect the severity of RV dysfunction and haemodynamic compromise in acute PE [62] • NT-pro-BNP levels have been shown to be elevated in a number of critical illnesses including sepsis, acute respiratory failure and major burns, and can provide prognostic information [63–65]	• NT-proBNP has a longer half-life compared with BNP. Hence levels of NT-proBNP are more stable and less influenced by acute haemodynamic variations	• The 95% percentile derived from a normal population: < 250 pmolL^−1^ (2118 pg mL^−1^) [65] • NT-proBNP < 400 pg mL^−1^ in an untreated person makes a diagnosis of HF less likely [60] • NT-proBNP > 400 pg mL^−1^ is considered elevated, and HF cannot be excluded. [60] • > 2000 pg mL^−1^ requires urgent referral for ECHO [60] • Values of ≈ 14000 pg mL^−1^ have been seen during critical illness [63]
**Neurohumoral markers**	MR-proADM		Widely expressed in many tissues and organ systems, including cardiovascular, renal, pulmonary, cerebrovascular, gastrointestinal, and endocrine tissues	• MR-proADM concentrations provide strong prognostic information in patients with acute HF [66]. In the BACH trial, MR-proADM was superior to both BNP and NT-proBNP in predicting mortality in AHF within 14 days. MR-proADM also provided significant additive incremental predictive value for 90-day mortality when added to BNP and NT-proBNP [67] • MR-proADM shows strong predictive value in sepsis [68], severe localised infections [69], and different types of organ failure in critical illness showing greater value than the routinely used PCT and CRP [70]. MR-pro-ADM assessments may be valuable for monitoring COVID-19 disease severity and stratifying the risk of critical illness or death [71]	• MR-proADM and ADM are derived by post translational processing from the precursor preproADM. MR-proADM and ADM are secreted in equimolar amounts and thus MR-proADM directly reflects ADM serum levels • preproADM is stimulated by volume overload to maintain endothelial barrier function, hence the interest in MR-proADM in cardiac dysfunction • MR-proADM, is a more stable peptide, has a longer half-life, has no biological effects and no circulating protein binding [2]—The measurement of the MR-proADM reflects the ADM concentration allowing the determination of its actual functional secretion	• The 2.5 and 97.5 percentiles are reported as 0.26 and 0.51 nmol L^−1^ respectively [72] • Values of 0.1–12.6 nmol L^−1^, (median of 0.88) have been documented in a large- scale study on acute dyspnoeic patients [67] • Values of ≈ 6 nmol L^−1^ are seen in critically ill patients with sepsis [68] and COVID-19 [71]
	MR-proANP		Predominantly expressed in the right atrium and secreted during an atrial distension such as in cardiac dysfunction or HF	• High MR-proANP plasma levels have been associated with disease severity and outcome of critical illness, particularly VAP, sepsis septic shock [73–75], and can rapidly identify left ventricular systolic dysfunction in sepsis patients [76] • MR-proANP is superior to BNP and pro-BNP in predicting death in CF patients [77] • It has shown strong prognostic utility in AIS, independently predicting post-stroke mortality and functional outcome [78] • It has shown diagnostic value for AHF in patients with acute dyspnoea [67]	• In response to increased tension of the atrial wall, the active hormone ANP is secreted by splitting of its precursor into NT-proANP, and an active hormone ANP 99–126. NT-proANP is further cleaved into smaller amino acid fragments in vivo, and hence MR-proANP is the preferred detection site of this natriuretic peptide. This has a half-life of approx. 2 h, compared with approx. 5 min for ANP	• Normal range—3.5–61.7 pmolL^−1^ (median 18.5 pmolL^−1^) [79] • A value of 120 pmolL^−1^ has been reported as an optimal cut-off for diagnosis of AHF [67] • ICU patients: 2.1–3417.0 pmolL^−1^ (median 214.0 pmolL^−1^) [79]
	Copeptin			• Useful in combination with cTn to safely and effectively rule-out AMI on admission with the first blood sample [80] • However, since the introduction of hs-cTn, copeptin has been shown to provide very little additive value [81] • Circulating levels of copeptin at ICU admission independently predict mortality in critically ill patients [82, 83] • Copeptin levels correlate with markers of renal failure and metabolic disturbances in ICU patients [84] and correlate with severity of sepsis [85] and traumatic brain injury [86]	• Copeptin assays are of extremely limited application diagnostically or prognostically for cardiac dysfunction [87] • Copeptin varies according to plasma osmolality in normal individuals, making a normal reference range difficult to derive	• A cut-off of copeptin at 10 pmolL^−1^ is recommended to rule-out AMI in combination with a negative Troponin. A value of 10 pmolL^−1^ or above is considered a positive result [80] • However, copeptin is elevated in ICU patients (46.4pmolL^−1^) in general compared with controls (median 4.7pmolL^−1^) [84], raising to values > 170pmolL^−1^ in septic shock [85]

**Inflammatory markers**	IL-6			• Increased levels of IL-6 are associated with deteriorating functional class of HF and with worse outcomes and adverse cardiac remodelling in these patients [88, 89] • IL-6 has been proposed as a potential therapeutic target in HF [90] • IL-6 concentration, independent of the already established predictors, correlates with adverse cardiac events [77, 78] • IL-6 concentrations are an independent predictor of 30-day mortality in patients with AMI complicated by cardiogenic shock [91] • IL-6 is a well-established independent prognostic marker in patients with COVID-19 and sepsis [92]	• There is huge interest in introducing routine measurement of IL-6 into critical care, particularly since the IL-6 receptor became a therapeutic target in COVID-19 • IL-6 is elevated in a wide range of inflammatory conditions, stimulated via IL-1β and TNFα • Inflammation is a fundamental process in the pathophysiology of several cardiac complications, most notably HF, and hence IL-6 is an important marker	• Normal range: < 0.7 pg mL^−1^ [93] • Values up to 15 ng mL^−1^ are seen in HF [88] • Values upwards of 50,000 pg mL^−1^ have been reported in patients with septic shock (median 376 pg mL^−1^), with median values of 48 pg mL^−1^ and 50 pg mL^−1^ reported in COVID-19 and trauma patients respectively [94]
	CRP		Synthesised primarily in liver hepatocytes but is also produced by smooth muscle cells, macrophages, endothelial cells, lymphocytes, and adipocytes	• CRP is a useful prognostic indicator in patients with ACS—elevated CRP levels are independent predictors of CV death, AMI, and congestive HF [4] • hs-CRP is often used to predict outcome in patients with heart disease, such as in AMI and ACS [95] • Hs-CRP strongly correlates with cTnI and CK-MB in STEMI and NSTEMI [96] and its peak concentration is significantly related to ejection fraction [97] • Recently CRP has been shown to be a useful indicator of cardiac injury in patients with COVID-19 [98]	• CRP is the most widely used inflammatory marker in routine general clinical practice but is certainly not cardiac specific • Cardiac events such as AMI invoke a large inflammatory response, which contributes to myocardial repair	• CRP levels of < 1, 1–3, and > 3 mgL^−1^ correspond to low-, moderate-, and high-risk groups for future cardiovascular events [99] • Median CRP levels of approx. 12mgL^−1^ are observed during MI [97] • However, CRP concentrations in the region of 200mgL^−1^ have been reported in critically ill patients with sepsis [100, 101]
	TNFα		Widely expressed by numerous cell types, but in the heart, it is mainly located in vascular endothelial cells and cardiac resident mast cells	• This marker is certainly not cardiac specific and is elevated in a wide range of inflammatory conditions, particularly so in critically ill patients [102] • Elevated TNFα levels can produce LV dysfunction, cardiomyopathy, and heart failure; raised serum TNFα is seen in patients with these conditions [88, 103] • In patients with advanced heart failure, TNFα concentration is an independent predictor of mortality [88, 104]	TNFα has well established potent negative inotropic effects During myocardial ischemia, TNFα concentrations rapidly increase contributing to the development of contractile dysfunction [105]	• Normal range: 0.7 + 0.3 pg mL^−1^ [93] • Values of 1-10 pg mL^−1^ are seen in MI [103] and HF [88] • However, values in the region of 320 pg mL^−1^ have been demonstrated in sepsis patients on the ICU [102]
**Markers of extracellular matrix remodelling**	sST2		Cardiomyocytes, endothelial cells, fibroblasts	• In the initial phase of AMI, during which symptoms of left ventricle dysfunction have not yet appeared, release of sST2 may indicate ongoing left ventricle stretching and predict the development of left ventricle dysfunction, therefore useful from a diagnostic perspective [106] • sST2 measurement on ICU admission has been shown to be useful to identify patients with higher risk of developing adverse cardiac events during unit stay [106] • Multiple studies have shown sST2 to be an excellent discriminator of mortality in patients with HF and AMI, and also non-cardiac ICU patients [107, 108]. sST2 has also shown prognostic and diagnostic value in ARDS [109] • The prognostic value of sST2 is additive to natriuretic peptides and (in the case of chronic HF) to cTnT	• Released in response to ventricular stretching, vascular congestion and inflammatory and profibrotic stimuli • Currently, measurement of sST2 has not been approved in the clinical guidelines as a prognostic biomarker of adverse outcomes in AMI. However, in the guideline of HF, sST2 is recommended for additive risk stratification, especially in the acute phase • It has been suggested that current data on sST2 justifies its measurement at least on admission for acute HF, and at the planned discharge to identify those patients with sustained elevated levels. This may predict; 1) a prolongation of hospital stay 2) those who may require a more rapid up-titration of HF drugs (after hemodynamic stabilization) 3) requirement for the use of monitoring systems to detect pulmonary congestion [110]	• Normal range: 4—31 ng mL^−1^ for males, • 2—21 ng mL^−1^ for females [111] • In patients diagnosed with STEMI, sST2 values of ≈ 250 ng mL^−1^ are reported [106] • This has been shown to rise to values in the region of 2000 ng mL^−1^ in some ICU patients [108].
	Gal-3		Macrophages, neutrophils, endothelial cells, epithelial cells	• Used for risk stratification /prognosis rather than diagnostics, especially in HF with preserved ejection fraction • Shown to predict mortality and HF hospitalisation and to correlate positively with LV end-systolic and end-diastolic volumes [112, 113] • Associated with mortality and increased risk of incident HF [114]. • A predictor of mortality, ICU access and ARDS stratification in patients with COVID 19 acute respiratory failure [115]	• Directly induces pathologic remodelling of the heart—implicated in the development of cardiac fibrosis	• Normal range: 3.8–21.0 ng mL^−1^ (from Gal-3 analysis in 1092 healthy volunteers aged ≥ 55 years from the Biolmage cardiovascular risk study) [116] • Values in HF have been reported between 5.0 and 66.6 ng mL^−1^ (from Gal-3 analysis in 592 HF patients) [117] • Values of ≈ 80 ng mL^−1^ have been reported in critically ill patients [27]
	GDF-8 (Myostatin)		Primarily, skeletal muscle, but also cardiomyocytes	• Used for risk stratification /prognosis rather than diagnostics • Upregulated in the heart after volume overload • GDF8 correlates strongly with other biomarkers related to HF severity [118] • Shown to reflect the extent of myocardial damage during AMI, similar to peak cTnI [119]. • Shown to reflect the severity of CHF and predict adverse prognosis in CHF patients [120]. • Conversely, low myostatin levels are associated with lower survival rates on the ICU, and baseline myostatin serum levels are an independent prognostic marker for overall survival in critically ill patients [121]. Levels are further significantly reduced in ICU patients requiring mechanical ventilation compared with those not [121]	GDF-8 is a negative regulator of skeletal muscle mass. It is significantly upregulated in muscle wasting conditions independently of cardiac dysfunction. However is also considered to be instrumental in the skeletal muscle wasting phenomenon in heart failure, cardiac cachexia and general critical illness [122]	• Normal Range: 10–80 ng mL^−1^ (geo mean 43 ng mL^−1^) (as demonstrated in a group of 60 healthy controls) [118] • In CHF: 30–105 ng mL^−1^ (geo mean 63 ng/ml) as demonstrated in a group of 76 CHF patients) [118] • Interestingly, Myostatin levels are significantly lower (median 10 ng mL^−1^) in ICU patients compared with controls [121], reflecting the role of this marker in inflammation-induced cachexia
	GDF-15		Multiple cell types, including cardiomyocytes, adipocytes, macrophages, endothelial cells, and vascular smooth muscle cells	• Used for risk stratification /prognosis rather than in diagnostics • Can predict risk of CV death/HF [123], recurrent MI [124], as well as risk of bleeding in NSTE-ACS patients [124] • GDF-15 is raised in ICU patients in general compared with controls [125], but is raised further in sepsis [125], ARDS [126], PE [127] and CS [128] • Prognostic value has been shown to be independent of traditional risk factors such as previous MI, age, elevated levels of other cardiac biomarkers • GDF-15 correlates with manifestation of organ failure including renal and hepatic failure and is associated with disease severity (APACHE II and SOFA) [125]	• A stress responsive member of the transforming growth factor β superfamily	• Normal range: 0.1–1.2 ng mL^−1^ [129] • GDF15 levels of 1.2–1.8 ng mL^−1^ are considered moderately elevated; > 1.8 ng/L^−1^ are considered severely elevated [130, 131] • Median GDF-15 levels in ICU patients have been reported at 5.8 ng mL^−1^, raising to > 7 ng mL^−1^ in septic patients [125], > 10 ng mL^−1^ in ARDS [126] and > 40 ng mL^−1^ in acute PE [127]

*ACC*, American College of Cardiology; *ACS*, acute coronary syndrome; *ADM*, adrenomedullin; *AHA*, American Heart Association; *AIS*, acute ischaemic stroke; *AMI*, acute myocardial infarction; *APACHE II*, Acute Physiology And Chronic Health Evaluation II; *ARDS*, Acute Respiratory Distress Syndrome; *BNP*, B-type natriuretic peptide; *CK-MB*, creatinine kinase-myocardial band; *CRP*, C-reactive protein; *CS*, cardiogenic shock; *cTnI*, cardiac troponin I; *cTnT*, cardiac troponin T; *CV*, cardiovascular; *ECHO*, echocardiogram; *ED*, emergency department; *Gal-3*, Galectin-3; *GDF-15*, growth/differentiation factor-15; *HF*, heart failure; *H-FABP*, heart-type fatty acid-binding protein; *HFSA*, Heart Failure Society of America; *HS*, high sensitivity; *ICU*, intensive care unit; *IL-6*, interleukin-6; *MR-proADM*, Mid-regional-pro-adrenomedullin; *MR-pro-ANP*, mid-regional-pro-atrial natriuretic peptide; *LV*, left ventricular; *NSTEMI*, non-ST segment elevation myocardial infarction; *NT-proBNP*, N-terminal (NT)-pro hormone BNP; *PE*, pulmonary embolism; *RV*, right ventricle; *SOFA*, Sequential Organ Failure Assessment; *sST2*, soluble-suppression-of-tumourigenicity-2; *TNF*, tumour-necrosis factor; *VAP*, ventilator associated pneumonia

## Alternative methods for the detection of cardiac biomarkers

Let us first consider the non-electroanalytical methods for the detection of cardiac biomarkers. Currently there are commercially available analysers for cTn, for example the TnI-Ultra assay (ADVIA Centaur XP immunoanalyzer, Siemens Healthcare Diagnostics) and the cTnT assay (Elecsys TnT-hs, Roche Diagnostics). The cTnI assay can achieve detection in plasma as low as 0.006 ng mL^−1^ and spanning a range of 0.006–50 ng mL^−1^, whereas, the cTnT assay has a limit of detection (LOD) of 0.005 ng mL^−1^ and can detect its presence up to 50 ng mL^−1^. These lab-based methodologies have improved significantly, with the Roche Troponin T assay able to produce results in a single hour [132]. However, there is still a huge drive for portable, reliable, and low-cost devices. Several commercial PoC benchtop devices are also available including BioMerieux Vidas, Mitsubishi Pathfast, and Radiometer AQT90, but development of more portable, hand-held, low-cost devices is still warranted.

Due to the significance of the topic, a plethora of other sensing methodologies have been reported throughout the literature for the detection of CBs. As such there are numerous reviews tackling many topics and their application to the detection of CBs which we direct the reader towards. These include general CB biosensors [133–136], lab-on-a-chip devices [137], fluorescence [138], colourimetric [139] nanomaterial-based [140, 141], acoustic-wave [142], potentiometric [143], and optical [144] to name just a few. Additionally, there have been reviews for electrochemical strategies [145–147], which often highlight a small number of markers or cover multiple detection methods. Herein, we focus solely on electrochemical-based strategies, giving comprehensive coverage of the published literature for the detection of a wide range of clinically proven and emerging CBs.

## Current electrochemical/electroanalytical approaches to detect cardiac biomarkers

Now let us consider that the electrochemical detection of cardiac biomarkers is an area of huge interest, with a plethora of different and interesting approaches reported. As expected within the exciting field of biosensor development, there are numerous works that utilise very similar strategies to achieve their end-goal of the quantification of the target biomarkers. For example, the use of EDC (carbodiimide compounds)/NHS (N-hydroxysuccinimide) coupling to covalently attach a bio-recognition element (e.g. protein/peptide) to the electrode surface, the utilisation of Au–S bonding on gold electrodes or the electrodeposition of AuNPs are widely utilised. It is commonplace for almost identical systems to be reported, varying only the electrochemical method (cyclic voltammetry (CV), differential pulse voltammetry (DPV), electrochemical impedance spectroscopy (EIS) etc.), photoelectrochemical (PEC), electrochemiluminescence (ECL), nanomaterial used (metallic nanoparticles, graphene, carbon nanotubes etc.) or simply the target detected. As such, we have aimed to include a comprehensive survey of the literature for each biomarker discussed in the form of tables for each section, highlighting the electrodes used alongside any modifications, the recognition element used, target biomarker and electrochemical detection technique used along with the key analytical parameters and real sample matrix. We do this whilst highlighting some unique and novel advances in biosensor technology and in cases where the literature is too vast (for example cTnI, cTnT and myoglobin) we will focus predominantly on strategies presented in the last 5 years. The electrochemical biosensors utilise three key recognition elements; these are antibody, aptamer, or molecular imprinted polymers (MIPs) person-made mimics of antibodies. Antibodies are widely used in the pharmaceutical industry but can be expensive, have limited stability and require the use of animals. A new approach is to utilise aptamers, synthetic molecules that can be raised against any kind of target and can bind their target with an affinity similar to, or higher than antibodies. Aptamers are ~ tenfold smaller than antibodies and can be chemically modified at-will in a defined and precise way. They can be easily stored and delivered, an advantage over antibodies, can be reversibly heat-denatured, and have a high batch to batch reproducibility. More recently, MIPs have been developed. These are artificial, highly cross-linked polymeric receptors that are engineered towards the binding of specific target analytes. This binding interaction is facilitated by nanocavities that are disturbed throughout the synthesized polymeric network, reflecting the conformation and chemical functionalities of the imprinted molecule or species. Advantages over conventional antibodies include superior chemical and thermal stability, ability to tailor the MIP to the template, and low-cost [148]. Clearly, all three recognition elements can be used in the development of electrochemical biosensors, but the advantages of aptamers and MIPs over antibodies is clear. Despite this, their advantages are not being fully utilised.

### Markers of myocardial injury

#### Cardiac troponin T (cTnT)

**Fig. 1:**
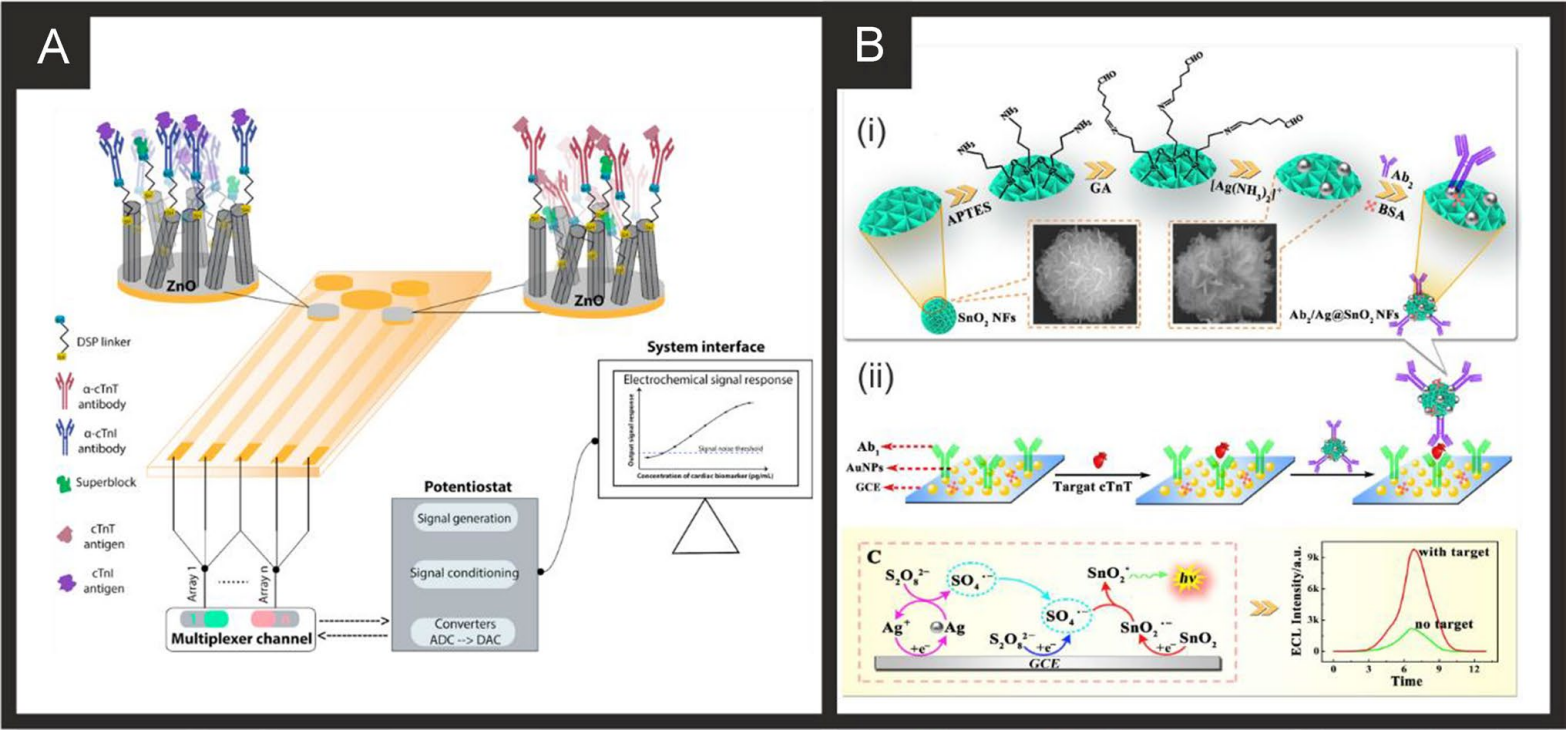
**A)** Schematic representation of sensing cTnI and cTnT biomarkers in a multiplexed sensor array format, utilising antibodies attached to ZnO nanorods. Reproduced with permission from ref [149]. Copyright Elsevier 2017. **B)** (i) Preparation process of the Ab_2_/Ag@SnO_2_ NFs signal probe; (ii) construction of the self-accelerated Ag@SnO_2_ NFs-based ECL immunosensor and (iii) proposed ECL mechanism for this system. Reproduced and adapted with permission from ref [151]. Copyright Elsevier 2018

The literature for this marker, alongside cTnI, is vast and we concentrate on the last 5 years only, which are summarised in Table 2. It is clear that immunoassay, aptamer and MIP based technologies are all being explored towards cTnT detection, producing clinically relevant linear ranges and detection limits with validation in predominantly human serum. Radha Shanmugam et al*.* [149] reported a multi-sensor immunoassay for cTnT and cTnI based on gold electrochemical platforms decorated with zinc oxide nanorods. Figure 1A shows a schematic overview of the sensing platform. In this approach, the multi-sensor is based upon thin film fabrication technology with a few nm ZnO seed layer deposited onto the working electrode via RF-Magnetron sputtering after which acts as nucleation sites for further hexagonal shaped ZnO nanorod growth when subjected to a low temperature hydrothermal bath consisting of a zinc nitrite salt and hexamethylenetetramine dissolved in water. The resultant morphology is the vertically oriented ZnO nanostructures, with their ends functionalised with an amine reactive crosslinker molecule—(dithiobis(succinimidyl propionate)), where the NHS ester group at its terminal end provides an amino-reactive surface that forms amine linkage with primary amine groups in the antibody molecule. The authors utilised electrochemical impedance spectroscopy (EIS) and Mott-Schottky analysis on the same sensor platform to demonstrate multi-configurable modes which allowed, via a “signal off” mechanism, the simultaneous measurement of cTnT and cTnI over the range of 0.1 to 1 × 10^5^ pg mL^−1^ with a LOD in human serum reported to correspond to 1 pg mL^−1^ for both cTnI and CTnT [149]. The authors report that ZnO is an attractive nanostructured material due to a high isoelectric point and high catalytic efficiency with the ability to align vertically the ZnO to provide a large surface area and useful attachment sites for the antibodies. The authors extended this to measure simultaneously cTnI, cTnT and BNP, showing the successful determination in human serum over the range of 1 pg mL^−1^–100 ng mL^−1^ with a LOD of 1 pg mL^1^ [150]. Jiang and co-workers [151] have developed an immunoassay sensor utilising electrochemiluminescence (ECL) via the fabrication of silver nanoparticles functionalized SnO_2_ nanoflowers where the latter are in the range of 1–2 µm fabricated via a facile hydrothermal methodology. The SnO_2_ nanoflowers were then functionalised with 3-aminopropyltrimethoxysilane (ATPES) by adding this dropwise into a solution containing the nanoflowers. These aminated nanoflowers were then dispersed into a glutaraldehyde solution to obtain aldehyde-terminated SnO_2_ nanoflowers. The nanoflowers were next dispersed into an ethanol solution to form a suspension with a silver ammonia solution added to obtain silver nanoparticle modified SnO_2_ nanoflowers via the traditional silver mirror reaction. To functionalise the Ag@SnO_2_ nanoflowers with the cTnT antibody probe, the former were simply mixed with the latter with an incubation of 12 h. Subsequently, bovine serum albumin (BSA) was added to the same solution to block the unspecificed nonspecific binding sites. The basis of the sensor is a sandwich type immunoassay with the second antibody attached to gold nanoparticles all supported upon a GCE. In the presence of cTnT, the sensor is a “signal on” where the ECL intensity is greater when the cTnT has binded between the two antibodies. The sensing approach requires 10 mM S_2_O_8_^2−^ in the solution that is measuring the target cTnT. The authors believe that the silver nanoparticles serve as a co-reaction accelerator which is able to react with the co-reactant of S_2_O_8_^2−^ for facilitating the ECL reaction between the SnO_2_ nanoflowers and S_2_O_8_^2−^ resulting in a stronger ECL signal compared with that of just the SnO_2_ nanoflowers in the presence of S_2_O_8_^2−^ (no silver nanoparticles). The immunoassay exhibited a large linear range from 1 fg mL^−1^ to 100 pg mL^−1^ with a LOD of 0.11 fg mL^−1^ reported. The immunoassay was shown to successfully determine cTnT in spiked human serum with recoveries in the range of 91.36 to 112.7%. Recently, Pourali and co-workers [152] reported a biosensing platform based on a sandwich immunoassay utilising CdS quantum dots (QDs). They developed a one-pot synthesis method for producing monodispersed CdS semiconducting nanocrystals (5 nm diameter), through the facile mixing of pre-cursers into a solvent system of dibenzyl ether and oleylamine. These nanocrystals were further modified with streptavidin and used for the signal enhancement. Detection was achieved through the binding of the CdS-streptavidin to biotinylated secondary antibodies followed by the use of square-wave anodic stripping voltammetry. Using this methodology, where the sensing mechanism is a “signal on”, the authors obtained a dynamic linear range from 5 to 1000 ng L^−1^ (0.005–1 ng L^−1^) with a detection limit of 2 ng L^−1^. The authors tested the effect of avidin, myoglobin and CK-MB, showing that the sensor retained at least 92% of its response. They further validated their results in human serum achieving RSD values of 9.8, 7.5 and 3.6% for three different fabricated immunosensors, additionally measuring recovery values between 95.6 and 105.1%.

**Table 2:** A summary of the reported literature for the electrochemical detection of the markers for myocardial injury; highlighting the marker(s) targeted, electrode materials and modifications, and the electroanalytical method used alongside the measured linear range, limit of detection and real sample medium

Cardiac biomarker	Electrode material	Sensor composition	Electroanalytical method	Dynamic range	Limit of detection	Real sample	Reference
cTnI, cTnT	Gold multiplex sensor	ZnO nanorods/DSP/Ab	EIS	0.1 pg mL^−1^–1 × 10^5^ pg mL^−1^	1 pg mL^−1^	Human Serum	[149]
cTnT	GCE	AuNPs-Hep/xAuNP/Ab	DPV	0.05–0.35 ng mL^−1^	0.016 ng mL^−1^	Blood Plasma	[153]
cTnT	SPE	Ab	CV	0–700 ng mL^−1^	0.15 ng mL^−1^	-	[154]
cTnT	GCE	AuNPs-Ab_1_/Ab_2_/Ag@SnO_2_ nanoflowers	ECL	1 fg mL^−1^–100 pg mL^−1^	0.11 fg mL^−1^	Human Serum	[151]
cTnI, cTnT and BNP	Gold multiplex sensor	ZnO nanorods/DSP/Ab	EIS	1 pg ml^−1^–100 ng ml^−1^	1 pg mL^−1^	Human Serum	[150]
cTnT	GCE	AuNPs/Ab_1_/ Ab_2_/CoS/ABEI-Ag	ECL	0.1 fg mL^−1^–100 pg mL^−1^	0.03 fg mL^−1^	Human Serum	[155]
cTnT	SPCE	AuNP/Ab/BSA	ECL	100 pg mL^−1^–5 fg mL^−1^	0.05 fg mL^−1^	Human Serum	[156]
cTnT	GCE	ZnSnO_3_/Ab	EIS	1 fg mL^−1^–1 μg mL^−1^	0.571 fg mL^−1^	-	[157]
cTnT	Gold	ZnO/DSP ZnO/APTES	EIS	10–300 pg mL^−1^	1 pg mL^−1^	Human serum	[158]
cTnT	Gold	NHS/EDC/PNIPAAm	CV/EIS	NR	NR	-	[159]
cTnT	GP	EDC/NHS/Ab	EIS/CV/SWV	0.5–1000 fg mL^−1^	1.28 fg mL^−1^	Human Serum	[160]
cTnT	Cr/Au	rGO/APTES/cTnT-Apt	RRC	1 pg mL^−1^–10 ng mL^−1^	1.7 pg mL^−1^	Human serum	[161]
cTnT	Gold	MGNS/cTnT-Apt/Ferrocyanide/MCH	DPV	0.05–5 ng mL^−1^	23 pg mL^−1^	Human Serum	[162]
cTnT cTnI Myo	Gold electrode array	Apt/CysA	ECL	0.50–4.0 ng mL^−1^ cTnT, 0.0010 -0.010 ng mL^−1^ cTnI, 0.050 -1.0 ng mL^−1^ Myo	0.30 ng mL^−1^ 31 pg mL^−1^ 0.79 pg mL^−1^	-	[163]
cTnT	SPCE	rGO/PPy MIP	DPV	0.01–0.1 ng mL^−1^	6 pg mL^−1^	Human Serum	[164]
cTnT	SPCE	rGO/c-PANI MIP	DPV	20–90 pg mL^−1^	8 pg mL^−1^	Human Serum	[165]
cTnT	SPCE	PMB/MWCNT/PANI MIP	DPV	0.1–8 pg mL^−1^	0.04 pg mL^−1^	Human Plasma	[166]
cTnT	Gold	*o*–PD/AAO MIP	LSV	0.04–0.2 ng mL^−1^	5.34 pg mL^−1^	Human Serum	[167]
cTnT	Gold	*o*–PD MIP	LSV	0.017–10 ng mL^−1^	1.7 × 10^−2^ ng mL^−1^	Blood serum	[168]
cTnT	SPCE	Ab_1_/Ab_2_/CdS/streptavidin	SWV	5–1000 ng mL^−1^	2 ng L^−1^	Human Serum	[152]
cTnI	Gold	fGQDs	CV	0.17–3 ng mL^−1^	0.02 ng mL^−1^	-	[169]
cTnI	GCE	Au nanorods/Ab1/BSA/Nitrogen/Sulfur-co-doped GO/L-lys/Au@Pt MBs/Thi	DPV	50 fg mL^−1^–250 ng mL^−1^, 750 fg mL^−1^–100 ng mL^−1^	16.7 fg mL^−1^	Human Serum	[170]
cTnI	GCE	Fe_3_O_4_-NH_2_/BSA/GLH/Co Pc NPs/Ab/APSM	AMP	1.0 pg mL^−1^–100 ng mL^−1^	0.39 pg mL^−1^	Human Serum	[171]
cTnI	GCE	CDs-3D-porous graphene-/Pd@Au nanocubes/Ab_1_/AuNPs/FMCS/Th/Ab_2_	AMP	1 × 10^−4^–100 ng mL^−1^	33.3 fg mL^−1^	Human serum	[172]
cTnI	GCE	PrGO/anti-cTnI	EIS	0.1–10 ng mL^−1^	0.07 ng mL^−1^	Bovine Serum	[173]
cTnI	GCE	G-MWCNT/Ab	EIS	1.0 pg mL^−1^–10 ng mL^−1^	0.94 pg mL^−1^	Human serum	[174]
cTnI	Graphene Chip	2-ABA/f-GN/Ab	LSV/EIS	0.01–1 ng mL^−1^	0.01 ng mL^−1^	Human Serum	[175]
cTnI	Gold	Ir(III) complex/Ab	EIS	1 ag mL^−1^–1 ng mL^−1^	10 ag mL^−1^	-	[176]
cTnI	SPGE	Disulfide-cored peptides	EIS	10–100 pg mL^−1^	1.9 pg mL^−1^	Serum	[177]
cTnI	Gold	AlGaN/GaN	EDL Gate	0.006–148 ng mL^−1^	2.62 pg mL^−1^	Human Serum	[178]
cTnI	GCE	AuNP/Peptide	EIS	0.016–1.55 ng mL^−1^	3.4 pg mL^−1^	Serum	[179]
cTnI	GCE	Ab/GCNT/PPCPPACP	EIS	1 pg mL^−1^–10 ng mL^−1^	1 pg mL^−1^	Human Serum	[180]
cTnI	Gold	PDDA-rGO/EDC/NHS/Ab	CV	0.1–10 ng mL^−1^	0.024 ng mL^−1^	Serum	[181]
cTnI	TiO_2_	CdS/PMSN/Cu^2+^/ssDNA	Photoelectrochem	1.2 fg mL^−1^—20 ng mL^−1^	0.47 fg mL^−1^	Human Serum	[182]
cTnI	ITO	Zn_2_SnO_4_/N,S-GQDs/CdS/TGA/Ab	Photoelectrochem	0.001—50 ng mL^−1^	0.3 pg mL^−1^	Human Serum	[183]
cTnI	Gold	DIL-HCNT/Ab	DPV	0.05–30 ng mL^−1^	0.02 ng mL^−1^	Bovine Serum	[184]
Myo cTnI CK-MB	GCE	MWCNT/SU-8/mAbs/EDC/NHS	EIS	1–50 ng mL^−1^ 0.1 -10 ng mL^−1^_,_ 10 ng mL^−1^- 10 µg mL^−1^	0.1 ng mL^−1^ 0.1 ng mL^−1^ 1 ng mL^−1^	-	[185]
cTnI	GCE	AuNC/GO/S-rGO/Ab	DPV	100 fg mL^−1^–250 ng mL^−1^	33 fg mL^−1^	Human Serum	[186]
cTnI	Gold	TI-Au-NS/Peptide	DPV	0.01–5 ng mL^−1^	0.9 pg mL^−1^	Human Serum	[187]
cTnI	Ti and Gold Plated Glass	Anti-cTnI M18/anti-cTnI M4/Protein G	CV/DPV	50 pg mL^−1^–1 µg mL^−1^	5 pg mL^−1^	Human Serum	[188]
cTnI	Gold	Fc-SiNPs/Tro4 Apt	SWV	0.024–240 ng mL^−1^	24 pg mL^−1^	Blood Plasma	[189]
cTnI	SPCE	AuNP/Tro4 apt/Tro6 apt hydrazine funct/TTCA	Chronoamperometry	0.024–2.4 ng mL^−1^	24 pg mL^−1^	Serum	[190]
cTnI	ITO	Mn_3_O_4_-rGO/cTnI-Apt	EIS	0.8–20 ng mL^−1^	0.8 ng mL^−1^	-	[191]
cTnI	Gold	ND-Au/cTnI-Apt	DPV	0.05–500 ng mL^−1^	8 pg mL^−1^	Blood Plasma	[192]
cTnI	Ti Foil	AuNP/cTnI-Apt	EIS	1–1100 pg mL^−1^	0.18 pg mL^−1^	Human serum	[193]
cTnI	SPCE	DNA-NTH/Tro4-Apt/Tro6-Apt/MMOF	DPV	0.05–100 ng mL^−1^	16 pg mL^−1^	Human Serum	[194]
cTnI	GCE	ZnONPs/MIP/Apt	EIS	1.25 × 10^–5^-8.25 µg mL^−1^	2.61 × 10^–5^ µg mL^−1^	Human Serum	[195]
cTnI	ITO	Ti_3_C_2_-MXene/AuNPs/T-DNA/Tro4-At/Au@Fe_3_O_4_	SWV	0.00239–23.9 pg mL^−1^	97 fg mL^−1^	Human Serum	[196]
cTnI	Gold	Apt/TdT/Mb-poly A	SWV	0.5–100 ng mL^−1^	0.04 ng mL^−1^	Human serum	[197]
cTnI	GCE	ZnONPs/PMB/Apt	EIS	0.012–7877 ng mL^−1^	25 pg mL^−1^	Human Serum	[195]
cTnI	GCE	*o*-AP	EIS	1.195–119.5 ng mL^−1^	0.65 ng mL^−1^	Human Serum	[198]
cTnI	GCE	BNQDs/PPy	DPV	0.01–5 ng mL^−1^	0.5 pg mL^−1^	Human Plasma	[199]
cTnI	GCE	AuNP-MWCNT/MIP/CS/GA	CV/DPV	0.005–60 ng mL^−1^	8 pg mL^−1^	Human Serum	[200]
cTnI	GCE	COOH-ZnONPs-Apt/MB MIP	DPV	0.012–7887 ng mL^−1^	0.02 ng mL^−1^	Human Serum	[201]
H-FABP	SPCE	*p*-Aminophenyl/Ab	AMP	4–250 ng ml^−1^	4 ng ml^−1^	Human Blood	[202]
H-FABP	Gold	EDC/NHS/Ab	EIS	0.098–25 ng mL^−1^	0.236 pg ml^−1^	-	[203]
H-FABP	Gold	mSAM/EDC/NHS/Ab/BSA	EIS	98 pg mL^−1^–100 ng mL^−1^	0.836 ng ml^−1^	Human Serum	[204]
H-FABP	GCE	AuNDs/Chit-g-Fc/Thi/PDA/OHCSs	DPV	0.001–200 ng mL^−1^	0.53 pg mL^−1^	Human Serum	[205]
H-FABP	GCE	Ni-TCPP (Fe)/PEI/Lum- /Ab_2_/BSA/Ab_1_/PICA	ECL	100 fg mL^−1^–100 ng mL^−1^	44.5 fg mL^−1^	Human Serum	[206]
H-FABP	GCE	Cd_0.5_Zn_0.5_S/d-Ti_3_C_2_T_x_ MXene/Ab_2_	DPV	0.01–1.00 pg mL^−1^	3.30 fg mL^−1^	-	[207]
H-FABP	ITO	rGO/NMIs/o–PD	DPV	1 fg mL^–1^–100 ng mL^–1^	2.29 fg mL^–1^	Human Serum, plasma	[208]
CK-MB	Gold	ThA/EDC/NHS/Ab_1_/AAP/Ab_2_	chronoamperometry	Up to 300 ng mL^−1^	13 ng mL^−1^	Human Serum	[209]
CK-MB	Au-SPE	Cysteamine/EDAC/NHS/Pcrea	SWV	0.19–28.8 µg mL^−1^	0.11 µg mL^−1^	Synthetic urine and serum	[210]
CK-MB	GCE	CNFs/MWCNTs/Ab	EIS	0.01–10 µg mL^−1^	1 ng mL^−1^	-	[211]
CK-MB	ITO	Avidin/BSA/Biotin-Ab_1_/Ab_2_/ALP/H_3_N-BH_3_/1A2N-P	chronocoulograms	100 fg mL^−1^–1 µg mL^−1^	80 fg mL^−1^	Human Serum	[186]
CK-MB	GCE	Cysteamine-GA-Cys/ creatine/Ab	DPV	0.1–2000 ng mL^−1^	0.04 ng mL^−1^	Human Serum	[212]
CK-MB	SWCNT-SPCE	CNO/Fe_3_O_4_/AuNPs/Chitosan/Ab/BSA/AgNPs/[Ru(bpy)_3_]^2+^	ECL	10 ng mL^−1^–50 fg mL^−1^	5 fg mL^−1^	Human Serum	[213]
CK-MB	GCE	AuPdCu nano-networks/Ab/BSA	Chronoamperometry	0.001–2000 ng mL^–1^	0.88 pg mL^–1^	Human Serum	[214]
CK-MB	GCE	PdPtCoNi@Pt-skin NPs/gold nano stars/thionine/Ab_2_/gold nano stars/Ab_1_	DPV	0.001–2500 ng mL^–1^	0.62 pg mL^–1^	Human Serum	[215]
CK-MB	Gold	Ti/Pd/CK Apt/EDC/NHS	EIS	0.1–100 ng mL^−1^	2.4 pg mL^−1^	Culture Medium	[216]
Myoglobin	SPCE	GQD/Ab	EIS	0.01–100 ng mL^−1^	0.01 ng mL^−1^	Serum	[217]
Myoglobin	SPCE	Cu doped ZnO NPs	EIS	51–255 ng mL^−1^	7.82 ng mL^−1^	-	[218]
Myoglobin	SPCE	Apt/GO/CNT	CV	1 ng mL^−1^–4 µg mL^−1^	0.34 ng mL^−1^	Bovine Serum	[219]
Myoglobin	SPCE	BP/PLL/Apt	CV	1 pg mL^−1^–16 µg mL^−1^	0.524 pg mL^−1^	Serum	[220]
Myoglobin	Gold	DApt-CS/Exo 1	CV/DPV	1.8–720 ng mL^−1^	0.49 ng mL^−1^	Human Serum	[221]
Myoglobin	ITO/Glass	PEI-rGO/Myo-Apt	DPV	0.001–1000 ng mL^−1^	2.1 pg mL^−1^	Human Serum	[222]
Myoglobin	Au-SPE	Polyphenol MIP	DPV	0.01 ng mL^−1^–100 µg mL^−1^	14 pg mL^−1^	Human Serum	[223]
Myoglobin	SPCE	*o*–PD	DPV	18–18,000 ng mL^−1^	9 ng mL^−1^	Human Plasma	[224]
Myoglobin	SPCE	Graphite/MIP	SWV	1.08–21.60 µg mL^−1^	0.79 µg mL^−1^	Urine	[225]
Myoglobin	GCE	MWCNT/PAPVIMBr	DPV	10.8–10,800 µg mL^−1^	0.175 µg mL^−1^	Human Serum	[226]
Myoglobin	Gold	3DG/PMMA	DPV	0.1 × 10^–10^-0.1 mg L^−1^	0.01 ng L^−1^	Horse Heart Standard	[227]
Myoglobin	GCE	MWCNT/Apt_1_/Apt_2_/MBPS	DPV	1.3 × 10^–8^-18000 ng mL^−1^	1.3 × 10^–8^ ng mL^−1^	Human Plasma	[228]
Myoglobin	CFME	Ab_1_/Ab_2_/ MoS_2_/CuS	CV	0.005–20 ng mL^−1^	1.2 pg mL^−1^	Human Serum	[229]
Myoglobin	Au-SPE	Mn-TiO_2_	CV	0.234–270 ng mL^−1^	0.234 ng mL^−1^	-	[230]
Myoglobin	rGO	Ab	EIS	0.09–180 ng mL^−1^	0.043 ng mL^−1^	Human Saliva	[231]
Myoglobin	SPE	AuNPs@rGO/Ab	DPV	1–1400 ng mL^−1^	0.67 ng mL^−1^	Human Serum	[232]
Myoglobin	ITO	g-C_3_N_4_-MoS_2_@CdS:Mn/Ab	Photoelectrochem	0.001—50 ng mL^−1^	0.42 pg mL^−1^	Human Serum	[233]

*cTn* cardiac troponin; *BNP* brain natriuretic peptide; *CK-MB* creatine kinase-myocardial band; *myo* myoglobin; *H-FABP* heart-fatty acid binding protein; *GCE* glassy carbon electrode; *SPCE* screen-printed carbon electrode; *CFME* carbon fibre microelectrode; *ITO* indium-doped tin oxide; *MWCNT* multi-walled carbon nanotubes; *rGO* reduced graphene oxide; *Ab* antibody; *DSP* dithiobis(succinimidyl) propionate; *AuNPs-Hep* heparin stabilised gold nanoparticles; *xGNP* exfoliated graphene nanoplatelets; *BSA* bovine serum albumin; *ABEI* N-(aminobutyl)-N-(ethylisoluminol); *APTES* 3-aminopropyl triethoxysilane; *NHS* N-hydroxysuccinimide; *EDC* N-ethylcarbodiimide; PNIPAAm: poly(N-isopropylacrylamide); *MGNS* microporous gold nanostructure; *MCH* mercaptohexanol; *Apt* aptamer; *CysA* cysteamine; *PPy* poly(pyrrole); *c-PANI* carboxylated poly(aniline); *PMB* poly(methylene blue); *o–PD o*-phenylenediamine; *AAO* anodic aluminium oxide; *MIP* molecularly imprinted polymer; *fGQDs* functionalised graphene quantum dots; *SU-8* epoxy-based negative photoresist; *MBs* magnetic beads; *Ths* thionine; *PEI* poly(ethyleneamine); *PMSN* positively charged mesoporous silica nanoparticles; *GLH* glutaraldehyde; *APMS* animated polystyrene microsphere; *FMC* functionalised mesoporous carbon; *2-ABA* 2-aminobenzyl amine; *f-GN* functionalised graphene; *GCNT* graphene carbon nanotubes; *PPCPPACP* poly(pyrrole-co-pyrrolepropylic acid); *PDDA* poly(diallylmethylammonium chloride); *DIL* dialdehyde functionalised ionic liquid; *Ti-Au-NS* triangular icicle gold nano structure; *PAA* poly(acrylic acid); ferrocene modified silica nanoparticles; *TTCA* 5,2’:5’,2’’-terthiophene-3’-carboxylic acid; *ND-Au* gold nanodumbells; *DNA-NTH* DNA nanotetrahedron; *MMOF* magnetic metal organic framework; *TdT* terminal deoxynucleotidyl transferase; *o-AP o*-aminophenol; *BNQDs* boron nitride quantum dots; *CS* chitosan; *PDA* polydopamine; *OHCSs* open pored hollow carbon spheres; *PICA* poly(indole-5-carboxylic acid); *ThA* thioctic acid; *AAP* ascorbic acid 2-phoshate; *EDAC* N-(3-dimethylaminopropyl)-N’-ethylcarbodiimide hydrochloride; *CNFs* carbon nanofibers; *ALP* alkaline phosphatase; *CNO* carbon nano-onions; *BP* black phosphorus; *PLL* poly(L-lysine); *DApt-CS* dual aptamer-complementary strand; *Exo 1* exonuclease 1; *PAPVIMBr* poly(1-[3-[(2-aminoethyl)amino]propyl]-3-vinylimidazole bromide); *3DG* 3-dimensional graphene foam; *PMMA* poly(methacrylic acid); *MBPS* methylene blue labelled polymersome; *EIS* electrochemical impedance spectroscopy; *DPV* differential pulse voltammetry; *CV* cyclic voltammetry; *ECL* electrochemiluminescence; *SWV* square wave voltammetry; *RRC* relative resistance change; *LSV* linear sweep voltammetry; *DL* electric double layer

Silva and co-workers developed a nano-molecularly imprinted polymer (N-MIP) assembled on reduced graphene oxide modified screen-printed graphite electrodes for sensing cTnT [164]. The biomimetic surface was obtained by first taking screen-printed graphite electrodes, which are then surface modified (via drop casting) with reduced graphene oxide; the authors attribute the use of reduced graphene oxide to improve electron transfer rates. The N-MIP was fabricated by taking the reduced graphene oxide screen-printed electrode and placing it into a solution containing cTnT, pyrrole and carboxylated pyrrole (COOH-3-Py) which is then electropolymerized via cyclic voltammetry. The authors explored a range of monomers in order to reach a maximal electron transfer; they also used organic polymers containing functional groups (carboxyl) in order to obtain more reactive biomimetic sites of the cTnT. The authors found that pyrrole and carboxylated pyrrole (COOH-3-Py) provided the best biomimetic conductive polymer where the carboxylic group in position 3 at the monomeric ring linked to the carboxylic group allowed the promotion of more interactions between reactive sites with cTnT. The authors justified their use of reduced graphene oxide in order to increase the synergy with PPy to increase electron transfer rates and promote greater numbers of biomimetic sites due to the nanostructured electrode surface area [164]. A critical parameter for N-MIPs is determining the dissociation constant, ${K}_{D}$ which can be calculated using a Langmuir isotherm model: ${I}_{CD}=\frac{{I}_{max}}{1+(\frac{{K}_{D}}{S})}$ where ${I}_{CD}$ is the current density, *S* is the concentration of the target (cTnT), and ${I}_{max}$ is the maximum current density. In terms of the N-MIP towards cTnT, a ${K}_{D}$ of 7.3 x 10^−13^ mol L^−1^ was found compared to the control (N-NIP) of 11.6 x 10^−13^ mol L^−1^ reflecting a high affinity of the biomimetic sites to low cTnT concentrations. The authors noted that the ${K}_{D}$ value of the N-MIP is comparable to that of conventional antibodies that exhibits *K*_D_ in the range of 10^−7^–10^−9^ mol L^−1^ [164] justifying their experimental development. The N-MIP modified electrodes were found to detect cTnT over the range 0.01 to 0.1 ng mL^−1^ with a very low LOD (0.006 ng mL^−1^) found to be possible using DPV. The authors went further and examined the N-MIP modified electrodes in human serum comparing their response with gold-standard ECLIA assays with recoveries found over the range of 97–115%. Phonklam et al. [166] followed a similar approach for the sensing of cTnT using MIPs upon screen-printed carbon electrodes with multi-walled carbon nanotubes modified via electrodeposition with the redox probe polymethylene blue. The authors reported that the use of carbon nanotubes increased the electrode area with a three-fold increase in the peak current/signal compared to the case of a bare electrode surface. The MIP was formed via the electropolymerization of polyaniline with the sensing mechanism based upon the redox probe polymethylene blue, where the binding of the cTnT with the MIPs impedes the electron transfer of the oxidation current providing a “signal off” sensor. The sensor was found to detect cTnT over the range of 0.10–8.0 pg mL^−1^ with a LOD of 0.040 pg mL^−1^ using DPV. The MIP sensor exhibited an excellent binding affinity (*K*_*D*_ = 2.8 × 10^–13^ mol L^−1^) comparable to others formed via different fabrication strategies and found that the sensor retained more than 90% of the sensitivity after 6 weeks of storage at room temperature. The authors determined cTnT in spiked human plasma which was found to compare well with an independent electrochemiluminescence method. MIPs clearly are an active range for sensing cTnT and from inspection of Table 2 we can see a range of MIPs [164–168] all evaluated in real samples and providing linearly useful analytical ranges. When considering application of such technologies in the ICU setting, we must be mindful of the analytical ranges needed, since these are typically developed with AMI “rule-out” in the emergency setting in mind; typical concentrations of cTnI in ICU patients may be in the region of 1000–1500 ng L^−1^ with many technologies being capable of translation into this setting [36].

#### Cardiac troponin I (cTnI)

**Fig. 2:**
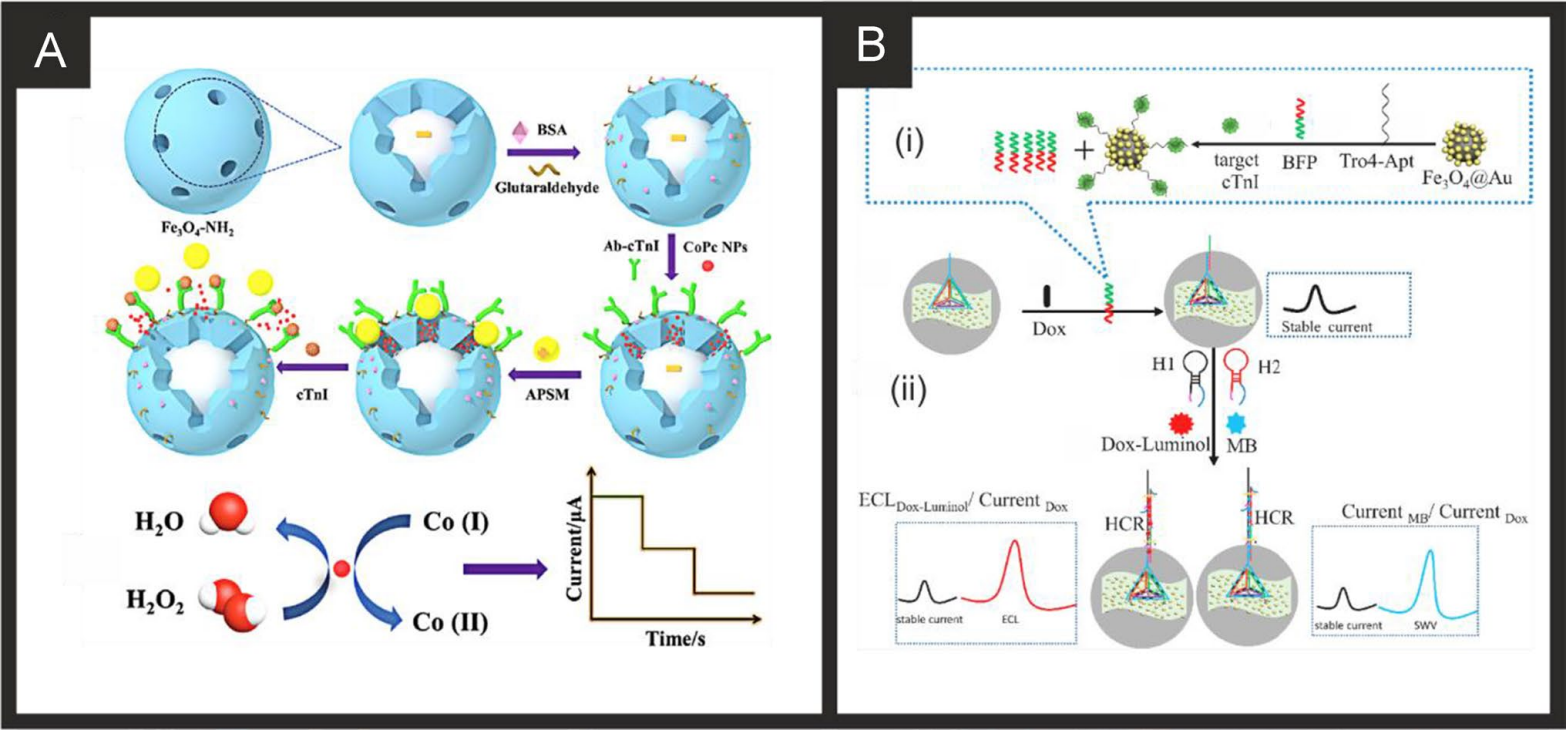
**A)** Preparation procedure for the sandwich-type electrochemical cTnI immunosensor based on mesoporous Fe_3_O_4_. Reproduced with permission from [171]. Copyright Elsevier 2019. **B)** An illustration of (i) the specific target recognition and BFP release, and (ii) the ratiometric biosensing mechanism for cTnI using an MXENE based sensor. Reproduced and adapted with permission from [196]. Copyright Elsevier 2021

From inspection of Table 2, a range of approaches have been reported utilising nanomaterials, such as using acetic acid functionalized graphene quantum dots (fGQDs) for an antibody free approach with a reported linear range of 0.17–3 ng mL^−1^ and a LOD of 0.02 ng mL^−1^ [169]. While the mechanism is attributed to hydrogen bonding interactions mediated by the carboxylic group in the fGQDs, the sensor is of limited use, if any, due to the lack of tests on real samples. As can be seen in Table 2, a large majority evaluate their sensor in real samples (human serum) which is a must for the credibility of any sensor. Ma and co-workers have reported an electrochemical immunoassay for the sensitive monitoring of cTnI using a novel controlled release system-based antigen-response [171]. Figure 1B shows a schematic overview of the electrochemical based immunosensor which is based upon Fe_3_O_4_-NH_2_ nanospheres (mean diameter of 150 nm) produced via a one-step solvothermal methodology. The nanospheres are mixed with glutaraldehyde (GLH) to provide functionalisation sites and the cTnI antibody and incubated for 2 h. Following this, bovine serum albumin (BSA) is used to block remaining active sites. Next, the surface is modified with the cTnI antibody and cobalt phthalocyanine nanoparticles (8-10 nm diameter). Aminated polystyrene microspheres (APSM) are then used to cover the mesoporous negative charged Fe_3_O_4_-Ab by electrostatic adsorption. As cTnI is introduced/analysed, APSM is separated from the Fe_3_O_4_ nanospheres which also releases the cobalt phthalocyanine nanoparticles. These latter released nanoparticles catalyse the added hydrogen peroxide (see Fig. 1B) and provide the electroanalytical signal via a “signal on” approach. This immunoassay was able to measure cTnI from 1.0 pg mL^−1^ to 100 ng mL^−1^ with a LOD of 0.39 pg mL^−1^ using amperometry. The authors went further and demonstrated their biosensor to measure cTnI in human serum with good recoveries (96.7–98.9%) and validated the proposed bioanalytical approach with ELISA indicating the biosensor to have a high accuracy and potential for clinical uptake. Mi et al*.* [196] have reported a ratiometric aptamer based sensing approach based upon the ECL signal of doxorubicin (Dox)-luminol or the electrochemical (EC) signal of methylene blue (MB) *vs.* referable EC signal of Dox. Figure 2 shows a schematic overview of the ratiometric aptamer sensor which utilises Ti_3_C_2_-MXene nanosheets fabricated by ultrasonic exfoliation resulting in 2 nm thick sheets indicating that they are few or single layer. The MXene nanosheets are then modified with gold nanoparticles and tetrahedral DNA (capture probe) which is combined with Au@Fe_3_O_4_ nanoparticles modified with Tro4-aptamer. In this approach, when cTnI binds with the aptamer, BFP (DNA sequence) is released, which hybridizes with the capture probe. A ECL luminophore (Dox-luminol complex) prepared by the cross-linking between Dox and luminol is used to amplify the ECL signal. Alternatively, the electrochemical signal of methylene blue can be used as an indicator allowing the sensor to be used as *ECL*_Dox-luminol_/*Current*_Dox_ or *Current*_MB_/*Current*_Dox_ (see Fig. 2). The approach provides a highly useful calibration signal (stable current signal, see Fig. 2) which increases the accuracy of detection which occurs via a “signal on” approach. The sensor is shown to be able to measure cTnI over the range 0.1 fM to 1 pM (0.00239–23.9 pg mL^−1^) with a LOD of 0.04 fM (0.97 fg mL^−1^). The authors demonstrated the sensor to measure cTnI in human serum and validated the measurements with ELISA which provided excellent agreement suggesting the sensor could be routinely used for the clinical measurement of cTnI.

**Fig. 3:**
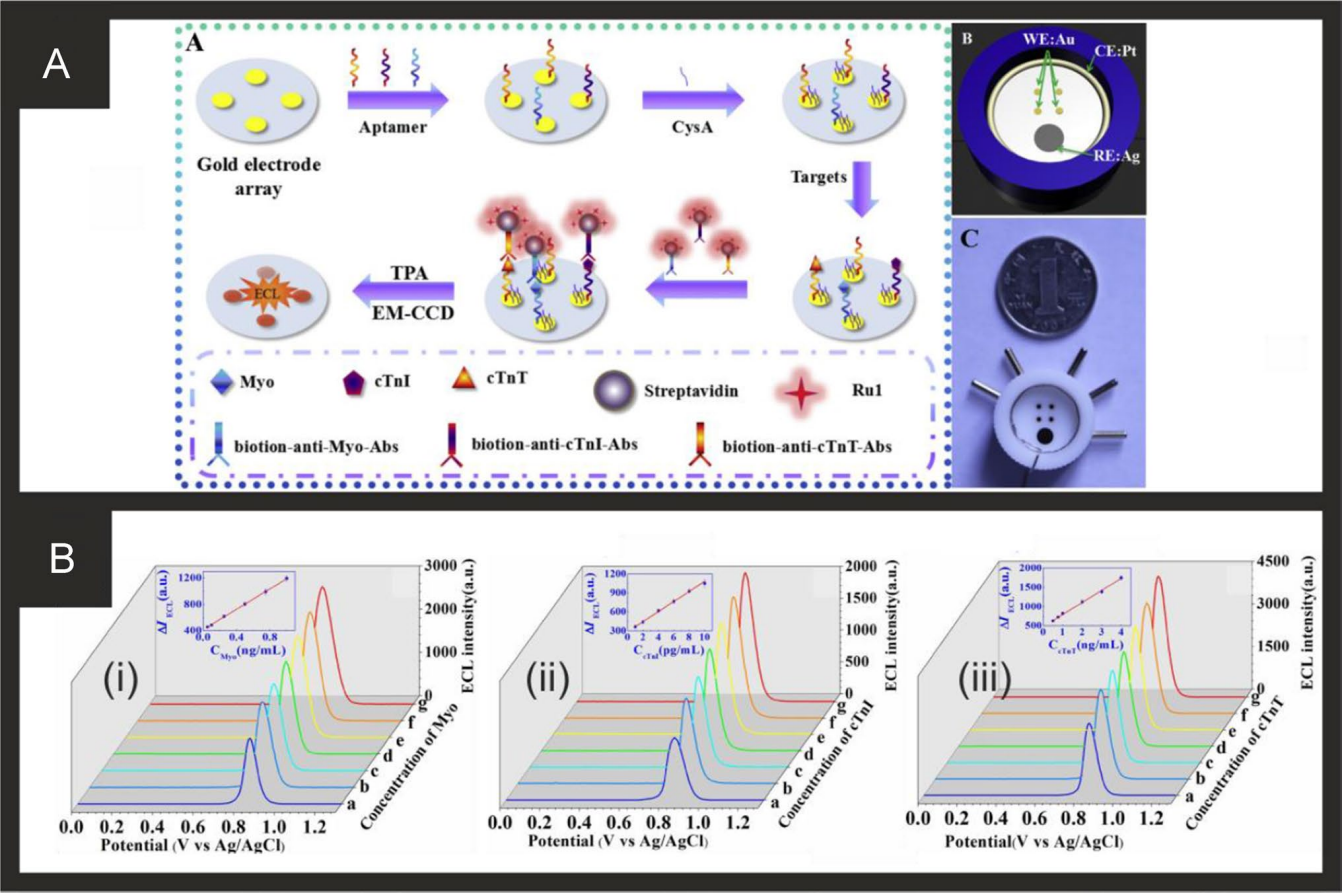
**A)** (i) Schematic diagram of the ECL biosensor array for the detection of three targets. (ii) Diagram and (iii) photograph of gold electrode array. **B)** ECL intensity-potential profiles for the determination of different concentration of myoglobin (i), cTnI (ii) and cTnT (iii). In (i): (ng/mL): (a) blank, (b) 0.050, (c) 0.10, (d) 0.25, (e) 0.50, (f) 0.75, (g) 1.0; In (ii) (pg/mL): (a) blank, (b) 1.0, (c) 2.0, (d) 4.0, (e) 6.0, (f) 8.0, (g) 10.0; In (iii) (ng/mL): (a) blank, (b) 0.50, (c) 0.75, (d) 1.0, (e) 2.0, (f) 3.0, (g) 4.0. Insert, calibration curve of Myo, cTnI and cTnT. Measurement conditions: 0.1 M PBS (pH 7.4) containing 50 mM TPA at a scan rate of 50 mV/s. Reproduced and adapted with permission from ref [163]. Copyright Elsevier 2018

Of note, Yang and co-workers [163] have utilised ECL for the simultaneous measurement of cTnT, cTnI and Myoglobin. Figure 3 shows the aptamer-based system and how the sensor is fabricated. The biosensor is based upon a gold macroelectrode array (2 mm diameter) which is modified with the cTnT, cTnI and Myo ssDNA aptamers and then with cysteamine (CysA). The sensor is then exposed to the analyte targets (cTnT, cTnI and Myoglobin) for 60 min which is then modified with a solution of biotinylaed antibody and the ECL probe, a ruthenium complex-labelled streptavidin (Ru1-SA). The ECL signal is based upon Ru(bpy)_3_^2+^-tripropylamine (TPA) undergoing electron transfer at the electrode surface to form an excited, light emitting state; see Fig. 3. Through the use of an Electron Multiplying Charge Coupled Device (EM-CCD), the ECL intensity-potential profiles are obtained providing the analytical signal. The multi- sensor was able to measure cTnT, cTnI and Myoglobin over the following linear ranges: 0.50–4.0 ng mL^−1^, 0.0010–0.010 ng mL^−1^, 0.050–1.0 ng mL^−1^ respectively with low detection limits of 0.30 ng mL^−1^, 0.79 pg mL^−1^ and 31 pg mL^−1^ respectively. Despite the achievement of excellent sensitivities, the dynamic ranges would need to be extended to be of use clinically, since the 99^th^ centile for cTnI for both men and women is outside of this range and given that concentrations of cTnI in critically ill populations can reach tenfold higher than the upper LOD reported here. The potential applicability of the sensor was shown to be viable in human serum samples with a commercial immunoassay. Again, it is important to consider the concentrations that may be seen in ICU patients to assess whether such technologies could be translated into this setting; it is recommended that a Myoglobin assay have a dynamic range of at least 500 ng mL^−1^ [56] and so some modifications would need to be made to achieve this. We would remind the reader, that although Myoglobin is generally considered an outdated CB for investigation of AMI/HF in emergency settings, very recent studies have shown its usefulness in sepsis and COVID-19 and have suggested it is superior to troponins in these settings [52–54]. Singal and co-workers [174] reported a simple yet elegant approach using a 3-dimensional graphene-multi walled carbon nanotube (G-MWCNT) hybrid prepared using a one-step chemical vapor deposition method with acetylene as a precursor source. The G-MWCNT film was transferred onto a glassy carbon electrode and modified with the cTnI antibody, attached through a molecular bi-linker, 1-pyrene butyric acid N-hydroxysuccinimide ester (PyBuNHS). The sensor exhibited a linear range from 1.0 pg mL^−1^ to 10 ng mL^−1^ with a LOD of 0.94 pg mL^−1^ using EIS and was shown to be successful to determine cTnI in human serum.

Last, of particular note is work by Zhang and co-workers [172] who reported a complex electrode configuration but yet provided the basis of a sensor for the ultrasensitive determination of cTnI with a LOD of 33.3 fg mL^−1^ and a linear range from 1 × 10^−4^ to 100 ng mL^−1^. The sensor is a sandwich type sensor based upon the use of nanoparticles labelled with antibodies with the sensing mechanism occurring via a “signal on” approach. A glassy carbon electrode, gold nanoparticle and thionine decorated amino-functionalized microporous carbon spheres provide the sensor platform, while gold nanotubes decorated with palladium, which have an average size of 35 nm diameter, are modified with β-cyclodextrins functionalized with 3D-dimensional porous graphene. Both nanoparticle composites are modified with antibodies. The sensing mechanism is based upon the increased electrocatalytic reduction of H_2_O_2_ mediated by thionine, resulting in a sensitive and reliable sensor response. The sensor was shown to successfully measure cTnI in spiked human serum and was compared with ELISA. Recoveries of between 98.0% and 102.4%, RSD values ranging between 3.3% and 4.5% and the relative error (1.7% to 3.8%) between the proposed sensor and ELISA suggest that the fabricated immunosensor has potential for the clinical application for cTnI detection.

#### Heart-type fatty acid-binding protein (H-FABP)

**Fig. 4:**
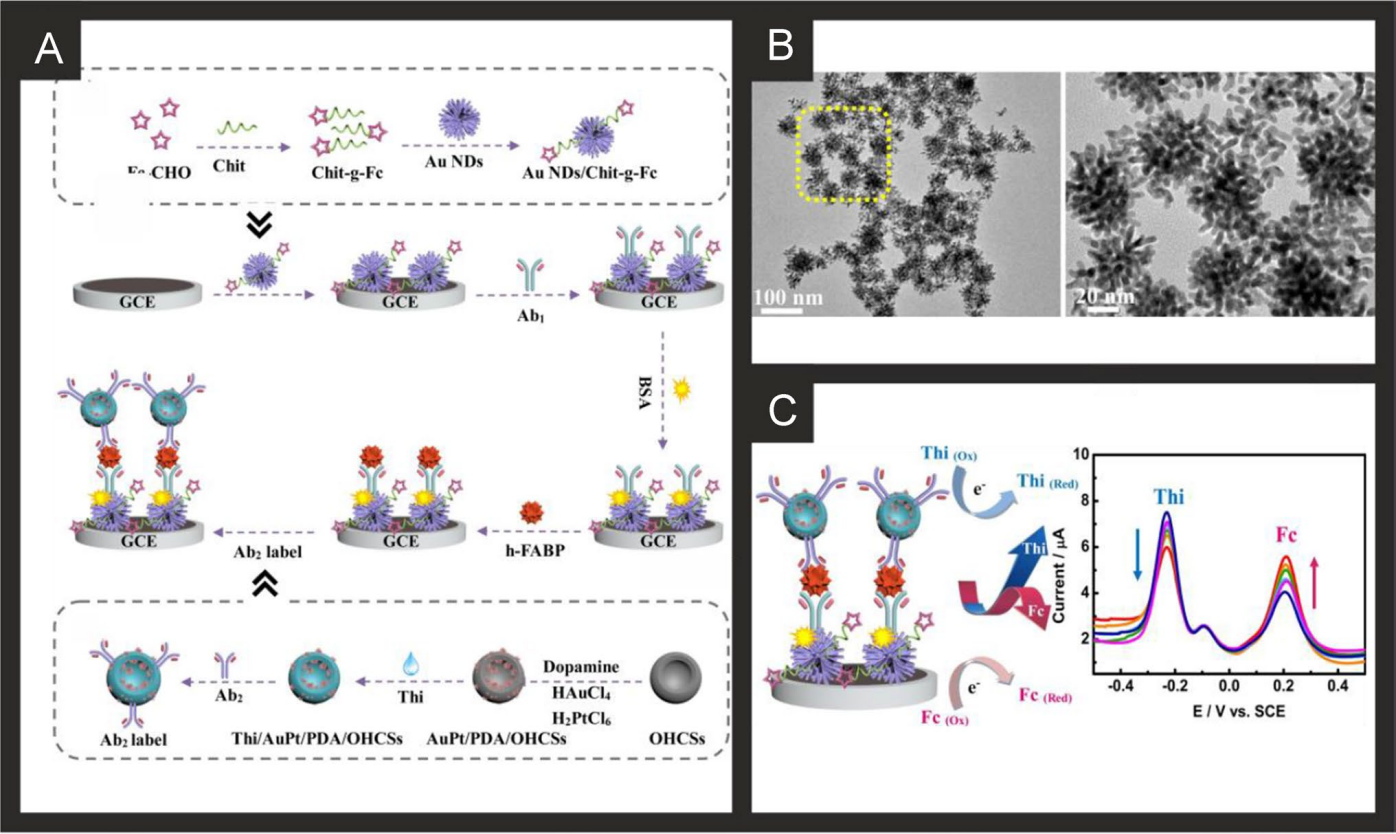
**A)** Schematic diagram for the construction of the ratiometric immunosensor for detecting H-FABP based on Au/Pt nanocrystals and open-pored hollow carbon nanospheres. **B)** TEM images of the gold nanodendrites. **C)** Summary of the electrochemical sensing platform and DPV signal acquired at 0.001, 0.01, 0.1, 1.0, 10 and 200 ng mL^−1^ of H-FABP. Reproduced and adapted with permission from ref [205]. Copyright Elsevier 2021

The first Sandwich Enzyme-Linked Immunosorbent Assay (ELISA) was reported by Ohkaru in 1995 [234] with the first electrochemical-based assay reported in 1996 [235] and many reported over recent years. Table 2 provides a summary of electrochemical based endeavours for the detection of H-FABP using a range of different and diverse nanomaterial sensing based platforms. Feng and co-workers have developed a ratiometric immunosensor for H-FABP; Fig. 4A,B shows a schematic diagram of the fabrication steps of the sensor and how it measures H-FABP [205]. The sensor utilises gold nanodendrites (Fig. 4C) synthesised by a simple methodology with ciprofloxacin hydrochloride coupled by attaching them onto chitosan-grafted-ferrocene prepared via a Schiff-base reaction, which are immobilised upon a GCE which acts as the substrate. The label material is based upon open-pored hollow carbon spheres (OHCSs) which have an of average diameter of 115.0 nm, which are modified with polydopamine (PDA), AuPt nanoparticles and thionine; see Fig. 4A, B. The OHCSs involve taking resorcinol dissolved into water after which PMMA nanospheres prepared via an emulsion polymerisation were added into the solution with formaldehyde and polyethylene glycol 600 (acting as a pore-forming agent) under stirring. The mixture was kept at 85 ℃ in the oven for 3 days, and elevated the temperature up to 800 ℃ for 2 h under a nitrogen flow. The large specific area and high porosity of this nanocomposite provides the efficient adsorption of the thionine electrochemical probe. Figure 4C, D shows a simplified image of the electrochemical sensing platform towards H-FABP and the resulting DPV curves from increasing concentrations of H-FABP. Note that ratiometric immunosensors have two “read outs”, i.e. two analytical signals with which to monitor the output of the sensor where the thionine and the ferrocene are both electroactive. This is a common approach to utilise these electrochemical redox probes in immunosensors and can potentially allow for self-calibration with improved sensitivity and accuracy over single-signal approaches. The sensor measures H-FABP over the range 0.001 to 200.0 ng mL^−1^ and has a very low LOD of 0.53 pg mL^−1^. The authors demonstrated the successful determination of H-FABP in human serum with recoveries of 100.1–101.7%, indicating that the sensor holds promise in clinical application [205].

Gan and co-workers [206] reported a highly sensitive electrochemiluminescence sandwich immunosensor for H-FABP determination based on a self-enhanced luminophore coupled with ultrathin 2D nickel metal–organic framework nanosheets. The nanosheets were synthesised via a surfactant-assisted methodology with the wrapping of PEI to produce an amino group to cross-link with luminol via glutaraldehyde which was then modified with H-FABP antibodies via gentle stirring overnight followed by adding BSA to eliminate nonspecific binding sites. The underlying ELC immunosensor is based upon a glassy carbon electrode (drop cast) modified with poly(indole-5-carboxylic acid) to increase the surface area with improved conductivity. The modified electrode was then immersed into a solution of NHS/EDC/MES for 12 h to activate the carboxyl group of the poly(indole-5-carboxylic acid). After this step, H-FABP antibodies were added by dropped solutions containing the antibody onto the electrode surface followed by the addition of BSA. This ECL immunosensor mechanism is based up on a “signal on” approach and was shown to exhibit a very wide detection range from 100 fg mL^−1^ to 100 ng mL^−1^ with an ultra-low LOD of 44.5 fg mL^−1^ and was shown to measure H-FABP in human serum with recover ranges from 98.7 to 102.7% with low % RSDs (4.2–8.4%). The authors ascribed the highly sensitive nature of the sensor to the following reasons: 1) the Ni-TCPP (Fe) nanosheets exhibiting good catalytic activity toward H_2_O_2_ decomposition but also acted as ideal nanocarrier for luminophore immobilization; 2) the use of a luminophore with a high stability, which shortens electron transport distance and reduce energy loss, effectively improving both the quantity and availability of luminol; 3) due to excellent conductivity and large surface area, poly(indole-5-carboxylic acid) (PICA) can facilitate electron transfer and significantly increase the immobilization amount of antibodies for further improvements in sensitivity [206]. The authors did comment that the conductivity of the Ni-TCPP (Fe) nanosheets is relatively low compared with noble metal nanomaterials, such that further improvement could be made through their incorporation in future sensors. Very recently, a MXene (Cd_0.5_Zn_0.5_S/d-Ti_3_C_2_T_x_) composite as signal amplificator and core–shell high-crystalline graphitic carbon nitride@carbon dots as electrochemical sensor platform have been utilised as the basis of an sandwich type immunosensor which operates via a “signal on” approach [207]. The MXene was prepared by subjecting a Ti_3_AlC_2_ MAX phase to etching in HCL/LiF for 20 h following ultrasonic treatment and centrifugation, delaminated MXene was obtained (d-Ti_3_C_2_T_x_). The d-Ti_3_C_2_T_x_ was added to an aqueous solution containing zinc and cadmium acetate salts, thioacetamide and subjected to a hydrothermal treatment at 180 degrees for 20 h. Following centrifugation, Cd_0.5_Zn_0.5_S/d-Ti_3_C_2_T_x_ is collected. This composite is then modified with H-FABP-antibody via magnetic stirring. The supporting electrochemical sensor platform was fabricated via a lengthy process which starts with carbon dots (CDs) being formed via the reaction of citric acid and ethylenediamine being heated at 250 degrees for 6 h and after cooling, impurities were removed via dialysis for 70 h. The CDs are then combined with a Ni foam template and dicyandiamide with crystallisation performed at 75 degrees for 15 h and then treated in a muffle furnace at 600 degrees for 90 min. The nickel foam is removed via treatment with 10 M acid with finally obtaining high-crystalline graphitic carbon nitride@carbon dots. These are then modified onto a glassy carbon electrode, coupled with the H-FABP antibody via drop casting, followed by the application of BSA. The immunoassay exhibited a linear range from 0.01 to 1.00 pg mL^−1^ with a LOD of 3.30 fg mL^−1^ [207] using DPV. While the immunosensor was shown to be highly selective in model solutions against 10 competitive proteins, with a single sensor shown to be able to be used over 50 times, no real samples were considered [207].

Last, as noted above, the detection of H-FABP is limited and generally based upon immunoassays, with very limited reports using MIPs [208]. For example, Sanati et al. [208] reported the development of a MIP based biosensor, based upon ITO modified electrochemically reduced graphene oxide (ERGO). These were modified with highly active surface area core–shelled gold nano/micro-islands (NMIs) via electrodeposition, which allows their size to be tuneable via controlling the electrodeposition process. The MIPs were fabricated via the electropolymerisation of *ortho*-phenylenediamine using CV in the presence of the target H-FABP. The MIP biosensor mechanism proceeds via a “signal off” and using DPV, exhibited a linear range from 1 fg mL^–1^ to 100 ng mL^–1^ towards H-FABP with a LOD of 2.29 fg mL^–1^ which was attributed to the high surface area of the NMIs and ERGO [208]. The sensor demonstrated two key aspect of MIPs that makes them attractive as the basis of electrochemical sensors, namely, stability and selectivity. In the former, the authors demonstrated the MIP biosensor was stable after 21 days of storage with only an 8.4% decrease in the electrochemical response. In the latter, the authors explored the interference of proteins found in human serum (e.g. albumin, globulin, and fibrinogen) with no effect and also myoglobin (*M*_W_ = 17.67 kDa) and troponin T (*M*_W_ = 35 kDa) were found to have no determinantal affect upon the sensor [208].

In summary the electrochemical based sensing strategies to the determination of H-FABP are on first sight, rather limited with all based upon immunoassay technology but have the downside of having multiple components which might be hard to implement in a commercial device. That said, the majority have been shown to successfully determine H-FABP in human serum/blood samples and future work should be used to extend the number of samples measured to produce clinically relevant information for uptake as a commercially device. With the exception of [234] and [203], the above mentioned sensors already cover the desired analytical ranges needed for assessment of this specific CB in critically ill patients [43]. The use of MIPs is very limited but are simpler in terms of the number of components needed to make a sensor and provide the successful measurement of H-FABP in human serum and plasma samples. Future work should be directed to developing new MIP based sensors.

#### Creatine kinase-myocardial band (CK-MB)

**Fig. 5:**
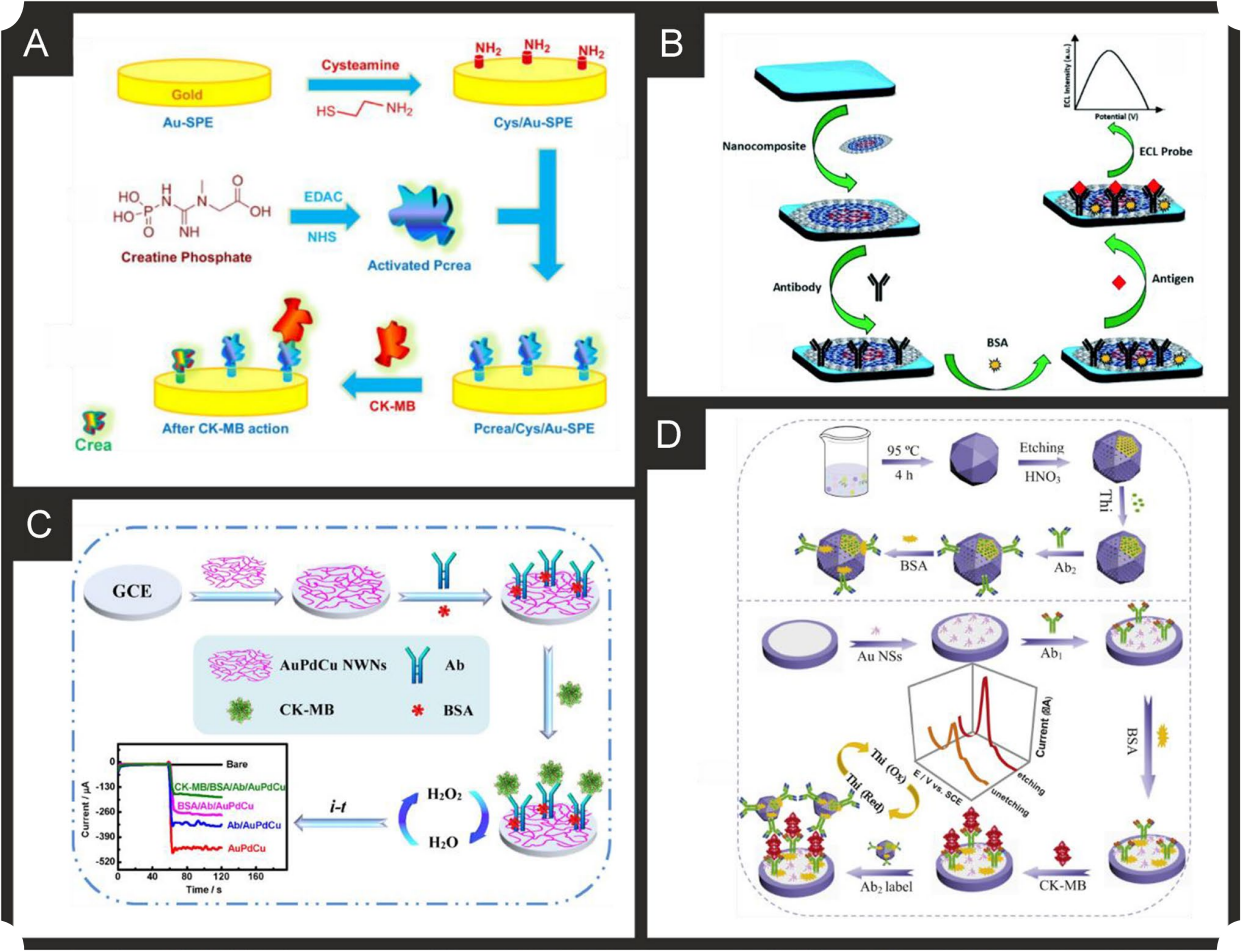
**A)** Schematic illustration for the stepwise preparation of the biosensor for CK-MB based on creatine phosphate. Reproduced and adapted with permission from ref [210]. Copyright Elsevier 2014. **B)** Schematic for the fabrication of the label-free ECL CK-MB immunosensor based on an CNOS/Fe_3_O_4_/AuNPs/CS modified SPE. Reproduced and adapted with permission from ref [213]. Copyright Royal Society of Chemistry 2019. **C)** Schematic illustration of the AuPdCu NWNs-based electrochemical sensor for detecting CK-MB. Reproduced and adapted with permission from ref [214]. Copyright Elsevier 2021. **D)** Schematic overview of the porous PdPtCoNi@Pt-skin nanopolyhedra production and their incorporation into an electrochemical immunoassay for CK-MB. Reproduced and adapted with permission from ref [215]. Copyright Elsevier 2020

Table 2 summarises various approaches to measuring CK-MB and on further examination, all are entirely focused upon immunoassays with none yet to utilise the potential benefits of aptamers and MIPs. The first electrochemical immunoassay was reported by Yuan and co-workers [236] using a platinum foil macroelectrode, anti-human CK-MB, NADH and ferricyanide, which provides the analytical signal. The authors demonstrated the successful determination of CK-MB in human serum and found a high correlation (0.999) with electrophoresis [236]. Moreira et al*.* [210] utilised gold screen-printed electrodes which are modified with a phosphorylated form of creatine (Pcrea). Figure 5A shows a schematic overview of the fabrication process where the gold SPE is modified with cysteamine and then via coupling Pcrea with *N*-(3-dimethylaminopropyl)-*N*'-ethylcarbodiimide hydrochloride (EDC) and *N*-Hydroxysuccinimide (NHS). As CK-MB binds to the Pcrea, it is monitored through the electrochemical response via SWV which results in a decrease (“signal off”) in the initial electroanalytical signal. The interferents cTnT, BSA, and myoglobin were studied with little effect on the electroanalytical signal and the authors found their sensor was able to measure CK-MB in synthetic urine and serum, [210] but clearly real samples are needed to progress the immunoassay. Li and co-workers [212] extended the work of Moreira et al*.* [210] using a GCE and demonstrated their immunoassay to work in human serum which compared favourably with an immunohistochemical staining method [212].

Adhikari and colleagues developed an ultra-sensitive label-free electrochemiluminescence (ECL) CK-MB immunosensor using a whole range of nanocomposite-modified single-walled carbon nanotube (SWCNT)-screen-printed electrodes (SPE) [213]. Figure 5B shows the schematic representation of the fabrication steps involved. A SWCNT-SPE is modified with a nanocomposite comprising carbon nano-onions (CNOs) that have been modified with Fe_3_O_4_ and gold nanoparticles (AuNP) and then chitosan (CS). All nanomaterials were commercially purchased with fabrication involving simple solution mixing, assembled via electrostatic interactions. The CNOs/Fe_3_O_4_/AuNP/CS composite was then drop casted upon the SWCNT-SPE. Next, the antibody-CK-MB is added onto the surface via drop casting, after leaving to incubate for 12 h, BSA is finally added onto the surface. The authors reported that the highly conductive behaviour of the nanoparticles with an increase in surface area due to the use of Fe_3_O_4_ and gold nanoparticles and the carbon nano-onions, may contribute to the enhanced ECL intensity. To monitor the binding of the CK-MB, the electrochemical probe [Ru(bpy)_3_]^2+^ and tri-n-propylamine (TPrA) were selected as the luminophore and co-reactant respectively, with the electrochemical mechanism described by the following:

$$\begin{array}{*{20}l} {\left[ {{\text{Ru}}\left( {{\text{bpy}}} \right)_{{3}} } \right]^{2+} - {\text{e}}^{-} \to \, \left[ {{\text{Ru}}\left( {{\text{bpy}}} \right)_{{3}} } \right]^{3+} } \hfill \\ {{\text{TPrA }} - {\text{ e}}^{-} \to {\text{TPrA}}^{\cdot +} } \hfill \\ {{\text{TPrA}}^{ \cdot +} \to {{\text{ TPrA}}^{\cdot}} \, + {\text{H}}^{+} } \hfill \\ \begin{array}{*{20}l} \left[ {{\text{Ru}}\left( {{\text{bpy}}} \right)_{{3}} } \right]^{{{3} + }} + {{\text{TPrA}}^{\cdot}} \, \to \, \left[ {{\text{Ru}}\left( {{\text{bpy}}} \right)_{{3}} } \right]^{3+} + {\text{ TPrA fragment}} \hfill \\ \left[ {{\text{Ru}}\left( {{\text{bpy}}} \right)_{{3}} } \right]^{2+\cdot} \, \to \, \left[ {{\text{Ru}}\left( {{\text{bpy}}} \right)_{{3}} } \right]^{{{2} + }} + {\text{h}}\nu\ ( \sim {62}0{\text{ nm}}) \hfill \\ \end{array} \hfill \\ \end{array}$$

The authors demonstrated that the label free ECL based immunoassay could detect CK-MB over the range 10 ng mL^−1^ to 50 fg mL^−1^ with a LOD of 5 fg mL^−1^ and the authors demonstrated the successful recovery of CK-MB from human serum with the recovery range between 98–103%. The use of screen-printed electrodes as the base electrochemical platform give potential for this to be up-taken commercially due to their low-cost, design flexibility and mass-producibility [237]. Although this sensor would be capable of detecting CK-MB values that fall outside of the normal range of 5 ng mL^−1^ [51], further work would be needed to allow concentrations in the range of 60–100 ng mL^−1^ in critically ill patients to be accurately reported.

Other reports have utilised nano-networks [214] such as an AuPdCu alloy fabricated by an eco-friendly one pot synthesis. The nano-networks are fabricated via a one-pot aqueous method where 4-aminopyridine along with gold, palladium and copper salts are mixed together with the reducing agent, ascorbic acid being added last, a process taking 3 h. The product was washed with water and centrifuged and dried in a vacuum. Figure 5C shows a schematic overview of the nano-network immunoassay, where the AuPdCu alloy nano-networks, which are of ~ 3 nm diameter, are drop-cast upon a GCE, which is then in turn modified with anti-CK-MB (via drop casting and 12 h incubation) and then BSA. The underlying mechanism is based upon the addition of hydrogen peroxide into the solution, which is electrochemically reduced at the alloy surface. In the presence of CK-MB which binds to the antibody, the electrochemical surface becomes inaccessible and a decrease (“signal-off”) in the electrochemical response is used as the analytical signal; Fig. 5C shows typical chronoamperograms. In using this approach, a linear range of 0.001 to 2000 ng mL^–1^ was shown to be possible with a LOD of 0.88 pg mL^–1^ reported and was demonstrated to be successful for the analysis of CK-MB in human serum with good recoveries (98.6–101.2%) with a RSD as low as 3.5%. Furthermore, the sensor could be stored for 28 days at 4 °C with only a 6.4% decrease in the electroanalytical signal. The authors attribute the sensor giving rise to the useful analytical performance to be due to the nano-networks providing a stable and large surface area and excellent biocompatibility for effectively capturing CK-MB [214].

Last, Wang and co-workers [215] have reported a sandwich type immunoassay for CK-MB detection which exhibited a linear range from 0.001 to 2500 ng mL^–1^ with a LOD reported to be possible 0.62 pg mL^–1^ using DPV. The sensor fabrication is shown within Fig. 5D where porous PdPtCoNi@Pt-skin nanopolyhedra particles (67.5–92.5 nm) are fabricated via one-pot aqueous approach and subsequent oxidation etching with nitric acid. These nanoparticles are then modified with thionine by dissolving the PdPtCoNi@Pt-skin nanopolyhedra particles into water under ultrasonication followed by the addition of thionine (Thi), stirred overnight. The Thi/PdPtCoNi@Pt-skin nanopolyhedra particles were then added into a phosphate buffer solution containing the CK-MB antibody, after which BSA was dropped into the same solution. The composite was obtained via centrifugation and washed. The electrochemical platform was produced by taking a GCE and drop casting gold nano stars, which are fabricated by dispersing thymine into water via ultrasonication until dissolved, accompanied by adding sodium hydroxide to adjust the pH to 11. Immediately after, a gold salt is added with the reducing agent ascorbic acid added dropwise with the reaction complete within 16 h. Onto this surface the CK-MB antibody was drop cast, modified with BSA and ready to use. In this sensor the thionine provides the electroanalytical signal via a “signal on” mechanism with which to indirectly measure the CK-MB. Figure 5D shows a typical DPV signal which demonstrates that the use of a chemical etching, with nitric acid gives rise to more porous PdPtCoNi@Pt-skin nanoparticles. The immunoassay was shown to determine CK-MB in human serum with good recoveries (99.2–101.0%) [215].

#### Myoglobin

Since the replacement of myoglobin with cTn’s as the biomarker of choice for the identification of MI, the number of reports on the development of new platforms for its detection have decreased. However, reports will continue to appear in the literature due to the far lower cost of purchasing myoglobin as a commercial analyte, making it the most accessible of the MI markers. The lower cost of myoglobin makes it especially attractive to the development of MIP based sensing platforms due to the large amount of variables needed to optimise (polymer composition, polymerisation methodology, ratios of target to monomer, template removal), in addition to the electrochemical parameters. Ribeiro and co-workers show these steps through the development of a myoglobin MIP based sensor using phenol as the MIP [223]. In this approach the MIP is made via the electrochemical polymerisation of phenol on a gold screen-printed electrode in the presence of Myo as the templating molecule using CV. The authors present the optimisation of the electrochemical parameters, template concentration, imprinting process, template extraction and analytical parameters. They performed their electrochemical oxidation of phenol at a neutral pH in order to facilitate the addition of proteins to the solution, settling on electropolymerisation of 10 mM phenol in the presence of 5 mg mL^−1^ myoglobin forming a polyphenol MIP with a thickness of ~ 4.4 nm similar to that of the Myo protein diameter. This high quantity of myoglobin required gives an indication of why researchers are hesitant to use a similar methodology with more expensive targets. Using this sensor, the authors achieved a dynamic range of 0.001 ng mL^−1^ to 100 µg mL^−1^, with a LOD of 2.1 pg mL^−1^ in buffer and 14 pg mL^−1^ in diluted artificial serum respectively using DPV. Recently, Farahani and co-workers have reported an ultra-sensitive electrochemical sensor for myoglobin based on aptamer recognition and methylene blue loaded co-polymers for signal amplification [228]. They explored the use of two types of poly(styrene)-block-poly(acrylic acid) amphiphilic co-polymers, both synthesised through reversible addition-fragmentation chain transfer polymerisation (RAFT), investigating their self-assembly into polymeric vesicles, as well as loading and release efficiency of the electroactive probe methylene blue. It was observed that the PS_61_-b-PAA_596_ provided greater loading and release capabilities for methylene blue. The biosensing platform worked through the immobilisation of an aptamer onto a gold surface, followed by incubation with myoglobin and further incubation with a secondary aptamer. The loaded polymersomes were then attached to the ends of the secondary aptamer through EDC/NHS coupling, followed by the addition of DMF to initiate the release of the methylene blue. The presence of methylene blue was detected using DPV in conjunction with a MWCNT modified GCE to produce a linear range of 1 aM to µM and a LOD of 0.73 aM; the sensing mechanism occurs via a “signal on”. They proceeded to show no interference from the presence of haemoglobin and acceptable recoveries in human plasma. It would be advantageous for this work to explore the effect of other common interferents toward myoglobin, in addition to validation of their real sample work through the use of commercial ELISAs.

An alternative detection methodology was presented by Ma and co-workers [233], who used photoelectrochemistry for the detection of myoglobin. They cast Mn-doped CdS nanocrystal-sensitized 2D heterostructured g-C_3_N_4_-MoS_2_ onto an ITO electrode to serve as the photoactive matrix. This heterostructured g-C_3_N_4_-MoS_2_ effectively promoted electron transfer and resisted the recombination of electron–hole pairs, producing a high photocurrent response, with the Mn-doped CdS further increasing the obtained photocurrent. This surface was modified with myoglobin specific antibodies to form the capture part of a sandwich assay. They used this in conjunction with anti-myoglobin labelled CuO conjugates, which effectively quenched the photoelectrochemical response of the system through competition for the light-generated electrons, poor conductivity and steric hindrance. Using this methodology, they were able to detect myoglobin in the range of 1 pg mL^−1^ to 50 ng mL^−1^, with a limit of detection of 0.42 pg mL^−1^. They exhibited that this system had a high specificity and sensitivity in human serum samples, achieving RSD% of 6.1% and below.

### Neuroendocrine markers and indicators of myocardial stretch

#### Brain natriuretic peptide (BNP)

**Fig. 6:**
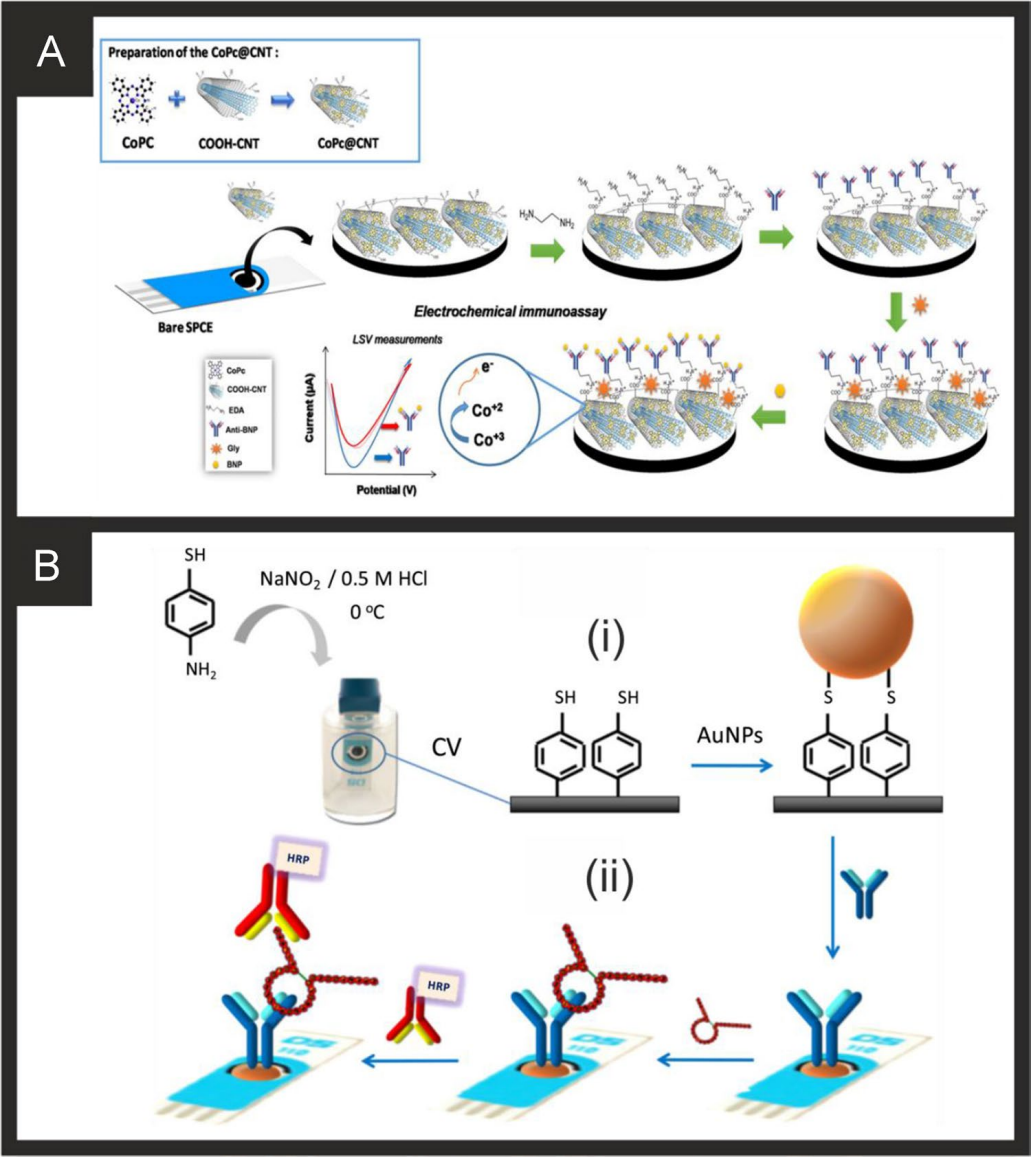
**A)** Schematic representation of the preparation and sensing of the CoPC@CNT based electrochemical immunoassay for BNP. Reproduced and adapted with permission from ref [238]. Copyright Wiley 2021. **B)** Schematic representation of the steps involved in the preparation of AuNPs-S-Phe-SPCEs (i) and HRP-anti-BNP-BNP-anti-BNP-AuNPs-S-Phe-SPCE immunosensor for the determination of BRP (ii). Reproduced and adapted with permission from ref [239]. Copyright Elsevier 2018

From inspection of Table 3, there are a limited amount of BNP sensors with the majority, if not all utilising immunoassay technology, and with very few using aptamer approaches. The approaches utilise a range of electrode compositions from acetylcholinesterase(AChE)-labelled anti-BNP gold nanoparticles, through to antibody labelled zinc oxide nanorods. Landim and co-workers [238] developed an immunosensor utilising screen-printed carbon electrodes (SPCE) which supported carboxylic acid functionalized multi-walled carbon nanotubes, modified with cobalt phthalocyanine (CoPc); see Fig. 6A. The electrode is then modified by drop casting with ethylenediamine (EDA), anti-BNP and glycine and left to react for 2 h. The immunosensor is based on the cobalt redox couple, which is the basis of the electroanalytical signal, with additions of BNP binding with anti-BNP, the signal decreases (“signal off”) due to the insulating nature of BNP antigen, blocking the kinetics of the interfacial electron transfer and preventing the electrochemical reduction of the CoPc, resulting in the decrease in the current [238]. The immunosensor was shown to measure BNP using LSV from 10 to 1000 ng L^−1^ with a LOD of 3 ng L^−1^, which is lower than conventional ELISA immunoassay for BNP quantification (14 ng L^−1^). The authors demonstrated their sensor to measure BNP in human serum with good recoveries (96–106%). Serafín and co-workers [239] reported an immunosensor based on the immobilization of capture antibodies onto gold nanoparticles (24 nm diameter, prepared from sodium citrate and gold salt) grafted on SPCEs through aryl diazonium salt chemistry, using 4-aminothiophenol (AuNPs-S-Phe-SPCE); Fig. 6B shows an overview of how the sensor is fabricated. Initially the electrodes are modified with 4-aminothiophenol via electrochemical grafting (via CV) onto which gold nanoparticles were immobilised, after which are then modified with the antibody via drop-casting and incubation for 30 min. Last, HRP-anti-BNP is immobilised onto the electrode surface and the sensor is ready. The sensor, was shown via amperometry to detect BNP over the range 14 to 15,000 ng L^−1^ with a LOD of 4 ng L^−1^ and was shown to successfully detect BNP in human serum and found to be in excellent agreement with ELISAs.

**Table 3:** Summary of the reported literature for the electrochemical detection of the markers for myocardial stretch, neurohumoral markers and markers of extracellular matrix remodelling; highlighting the marker(s) targeted, electrode materials and modifications, and the electroanalytical method used alongside the measured linear range, limit of detection and real sample medium

Cardiac biomarker	Electrode material	Sensor composition	Electroanalytical method	Dynamic range	Limit of detection	Real sample	Reference
NT-proBNP	Gold	Anti NT-proBNP	Amperometry	0.04–2.5 ng mL^−1^	0.03 ng mL^−1^	Human Serum	[240]
NT-proBNP	Gold	BSA-CNTs/ DpAu/ Ab_1_ / NT-proBNP/ Au NCs-HRP labeled Ab_2_	Amperometry	0.02–100 ng mL^−1^	6 pg mL^−1^	-	[241]
NT-proBNP	Gold	M-NPs / BAS/ anti-NT-proBNP	Amperometry	0.005–1.67 ng mL^−1^ 1.67–4 ng mL^−1^	0.003 ng mL^−1^	Human Serum	[242]
NT-proBNP	SPGE	HOOC-MBs EDC/sulfo-NHS/NT-proBNP/HRP-anti-NT-proBNP/TMB	Amperometry	0.12–42.9 ng mL^−1^	0.02 ng mL^−1^	Human Serum	[243]
NT-proBNP	Gold	NHS/EDC/anti-NT-proBNP	EIS	NR	10 fg mL^−1^	-	[244]
NT-proBNP	Gold	Anti-NT-proBNP	EIS	10–1000 pg mL^−1^	NR	-	[245]
NT-proBNP	GCE	PtNPs/ Ab_1_/BSA/anti-NT-proBNP and [Ru(dcbpy)_3_]^2+^ / LM-MOFs/AuNPs/Ab_2_/BSA	ECL	0.005–25 ng mL^−1^	1.67 ng mL^−1^	Human Serum	[246]
NT-proBNP	ITO	COOH-MWCNTs/ chitosan/ GNDs/ ABEI/ GA/ anti-NT-proBNP	ECL	0.01–100 pg mL^−1^	3.86 fg/mL − 1	Human Plasma	[247]
NT-proBNP	GCE	AuNFS/Ab_1_/BSA/ and PdCu@SWCNHs/PTCA-Lu/Ab_2_/BSA	ECL	0.1–25000 pg mL^−1^	0.05 pg mL^−1^	Human Serum	[248]
NT-proBNP	GCE	Au NPs@GO-Ru(bpy)_3_^2+^/Ag_2_C_2_O_4_-Ab_1_ and Fe_3_O_4_@PDA-Ab_2_	ECL	0.0005–100.0 ng mL^−1^	0.28 pg mL^−1^	Human Serum	[249]
NT-proBNP	ITO	SnO_2_/NCQDs/Bi_2_S_3_/TGA/EDC/NHS/Anti-NT-proBNP/BSA	Photoelectrochem	0.01–50 ng mL^−1^	3.7 pg mL^−1^	Human Serum	[250]
NT-proBNP	ITO	Au@ZnO/ 3D ZnIn_2_S_4_/ La-CdS/PDA/anti-NT-pro-BNP/BSA	Photoelectrochem	0.0008–45 ng mL^−1^	0.32 pg mL^−1^	Human Serum	[251]
NT-proBNP	GCE	Luminol-Au@Fe_3_O_4_-Cu_3_(PO_4_)_2_/Ab_1_/BSA and rGO-Au@CuS-Ab_2_	ECL	0.0005–20 ng mL^−1^	0.12 pg mL^−1^	Human Serum	[252]
NT-proBNP	GCE	Ti:BiOBr-Au/anti-NT-proBNP/BSA	ECL	0.001–50 ng mL^−1^	0.33 pg mL^−1^	Human Serum	[253]
NT-proBNP	ITO	SnO_2_/SnS_2_/mpg-C_3_N_4_/(EDC/NHS)/Ab_1_/BSA and PbS/SiO_2_-Ab_2_	Photoelectrochem	0.1–50000 pg mL^–1^	0.05 pg mL^–1^	Human Serum	[254]
NT-proBNP	GCE	AgNC-Sem@AuNP/Ab_1_/BSA and MIL-125/Ab_2_	ECL	0.00025–100 ng mL^−1^	0.11 pg mL^−1^	Human Serum	[255]
NT-proBNP	GCE	Ce-MOF@g-C_3_N_4_Au/anti-NT-proBNP/BSA	ECL	0.005–20 ng mL^−1^	3.59 pg mL^−1^	Human Serum	[162]
NT-proBNP	GCE	TiO_2_NanoFlowers@CN-Au/Ab_1_/BSA and PDA@Ab_2_	ECL	0.0001–10 ng∙mL^−1^	50 fgmL^−1^	Human Serum	[256]
NT-proBNP	GCE	Au/anit0NT-proBNP/BSA and HKUST-1/Ab_2_	ASV	5 × 10^−7^–500 ng mL^−1^	3.3 × 10^–4^ pg mL^−1^	Human Serum	[257]
NT-proBNP	GCE	MoS_2_@Cu_2_S/AuNPs/Ab_1_/BSA and MOF/Ab_2_	ECL	1 fg mL^−1^–100 ng mL^−1^	0.41 fg mL^−1^	Human Serum	[258]
NT-proBNP	SPCE	EDC/NHS– AB_1_ and AgNP /EDC/NHS– AB_2_	DPV	25–1000 ng mL^−1^	4.0 ng mL^−1^	Human Serum	[259]
NT-proBNP	Paper electrode	MgB-Ab_1_ and AgNP-HBCL-Ab_2_	ASV	49.3–198.05 ng mL^−1^	9.86 ng mL^−1^	Human Serum	[260]
NT-proBNP	GCE	PtCoNi HMBs/Fc-g-IL/BSA/Ab_1_ and Au NSs/thionine/Co–N-C nanosheets/Ab_2_	DPV	0.001–10.0 ng mL^–1^	0.35 pg mL^–1^	Human Serum	[261]
NT-proBNP	GCE	AuNP/CNTs	ECL	2.178–32159 pg mL^−1^	1.3 pg mL^−1^	Human Serum	[262]
NT-proBNP	GCE	DpAu-Pt/ SH-CBA1	ECL	0.01–500 ng mL^−1^	0.77 pg mL^−1^	-	[263]
NT-proBNP	Au-IDE	APTES-ZEO-IO/GLU/ Streptavidin/Biotin Apt	LSV	0.0085–0.272 ng mL^−1^	0.0085 ng mL^−1^	Human Serum	[264]
NT-proBNP	Au-IDE	APTES/GNR/Apt	LSV	0.001–100 ng mL^−1^	1 pg mL^−1^	-	[265]
BNP	Silver	AChE-anti-BNP/AuNPs	LSV	20–200 pg mL^–1^	20 pg mL^–1^	-	[266]
BNP	Silver	AChE-anti-BNP/AuNPs	LSV	NR	20 ng mL^–1^	Human Serum	[267]
BNP	SPCE	AuNPs/streptavidin/Ab/BSA/Anti-BNP-HRP	CV	10–100 fg mL^–1^	34 fg mL^–1^	-	[268]
BNP	SPCE	4-aminothiophenol/AuNPs/Ab/HRP-Ab	EIS	0.014–15 ng mL^−1^	4 pg mL^−1^	Human Serum	[239]
cTnT, cTnI and BNP	Gold	ZnO nanorods/Ab	EIS	0.001–100 ng mL^−1^	1 pg mL^−1^	-	[150]
BNP	SPCE	AuNPs/Thionine/NH2-Graphene/Ab	Amperometry	0.05–30 ng mL^–1^	0.012 ng mL^–1^	Human Serum	[269]
BNP	Gold	16-MHDA/EDC/NHS/Ab/ethanolamine	EIS	1–1000 pg mL^−1^	NR	Rabbit blood	[270]
BNP	GCE	GS/SnO_2_/PAN-Au/BNP-Ab and ZnCo_2_O_4_/N-CNTs-Ab	Amperometry	0.01–1000 pg mL^−1^	3.34 fg mL^−1^	Human Serum	[271]
BNP	Gold	CNTs/ Anti-BNP/BSA	EIS	0–4000 pg mL^−1^	16 pg mL^−1^,	Blood plasma	[272]
BNP	SPCE	CoPc@CNT/ EDA/ Anti-BNP/ Glycine	LSV	10–1000 pg mL^−1^	3 pg mL^−1^	Human Serum	[238]
BNP	ITO	N–ZnO NP- PPIX/BNP-Apt	Photoelectrochem	1 pg mL^−1^–0.1 μg mL^−1^	0.14 pg mL^−1^	Human Serum	[273]
BNP	ITO	CeO_2_/CdS/Ab_1_/Ab_2_/SiO_2_-PDA-Ag	Photoelectrochem	0.1 pg mL^−1^–5 ng mL^−1^	0.05 pg mL^−1^	Human Serum	[274]
Copeptin	ITO	RGO–TiO2/ EDC/NHS/Ab-copeptin/BSA	CV	249–12344 pmol L^−1^	37.3 pmol L^−1^	-	[275]
Copeptin	ITO	Cu^2+^-Cys-ABEI-AuNPs-Chitosen/Ab-GNPs /BSA	ECL	0.02–10 pmol L^−1^	0.0005 pmol L^−1^	Human Serum	[276]
Copeptin	GCE	TEOA@MOFs/GO/GA-Chitosan/anti-copeptin/BSA / Ru(bpy)_3_^2+^	ECL	1.24–12344 pmol L^−1^	0.09 pmol L^–1^	Human Serum	[277]
sST2	SPCE	MBs/Ab_1_/Ab_2_/streptavidin/HRP	Amperometry	141–2500 pg mL^−1^	39.6 pg mL^−1^	Human Plasma	[278]
sST2	GP	C_60_/Ab	EIS	0.1–100 fg mL^−1^	1.28 fg mL^−1^	Human Serum	[279]
Gal-3	GCE	AuNP@Fc-Lac	DPV	4.8–15 µg mL^−1^	4.8 µg mL^−1^	-	[280]
Gal-3	SPCE	MBs/Ab_1_/Ab_2_/streptavidin/HRP	Amperometry	0.028–5 ng mL^−1^	8.3 pg mL^−1^	Human Serum	[281]
Gal-3	GCE	CG/Ab_1_/Ab_2_/AuNP/MB/MSN	ASV	0.5 fg mL^−1^–500 ng mL^−1^	0.17 fg mL^−1^	Human Serum	[282]
Gal-3	GCE	N-GNRs-Fc-MOFs@Au/Ab_1_/Ab_2_/AuPt-MB	DPV	100 fg mL^−1^–50 ng mL^−1^	33.33 fg mL^−1^	Human Serum	[283]
Gal-3	SPCE	Aminophenol MIP	EIS	0.5 ng mL^−1^–500 µg mL^−1^	-	Human Serum	[284]

*NT-proBNP* N-terminal-pro b-type natriuretic peptide; *BNP* B-type natriuretic peptide; *sST2* soluble suppression of tumorigenesis-2; *Gal-3* galectin-3; *SPGE* screen-printed graphene electrode; *SPCE* screen-printed carbon electrode; *GCE* glassy carbon electrode; *ITO* indium-doped tin oxide; *Au-IDE* gold interdigitated electrode; *GP* graphite paper; *CV* cyclic voltammetry; *DV* differential pulse voltammetry; *LSV* linear sweep voltammetry; *EIS* electrochemical impedance spectroscopy; *ECL* electrochemiluminescence; *ASV* anodic stripping voltammetry; *Ab* antibody; *Apt* aptamer; *BSA* bovine serum albumin; *CNT* carbon nanotube; *M-NPs* magnetic nanoparticles; *BAS* biotin-avidin system; *MBs* magnetic beads; *EDC* N-ethylcarbodiimide; *NHS* N-hydroxysuccinimide; *HRP* horseradish peroxidase; *TMB* 3,3’,5,5’-tetramethylbenzidine; *PtNPs* platinum nanoparticles; *MOFs* metal organic frameworks; *MWCNTs* multi-walled carbon nanotubes; *GNDs* gold nanodots; *ABEI* N-(aminobutyl)-N-(ethylisoluminol); *GA* glutaraldehyde; *AuNFs* gold nanoflowers; *SWCNHs* single-walled carbon nanohorns; *PTCA* 3,4,9,10-perylenetetracarboxylic acid conjugated luminol; *GO* graphene oxide; *PDA* poly(dopamine); *NCQDs* nitrogen-doped quantum dots; *TGA* thioglycolic acid; *rGO* reduced graphene oxide; *mpg-C*_*3*_*N*_*4*_ mesoporous carbon nitride; *AgNC-Sem* semicarbazide-modified gold nanoparticles; *MOF* metal organic framework; *HKUST-1* Cu^2+^-1,2,5-benzenetricarboxylic acid metal organic framework; *HMBs* hollow multi-branches; *AuNS* gold nanostars; *APTES* 3-aminopropyl triethyoxysilane; *ZEO-IO* zeolite-iron oxide; *GNR* gold nanorods; *16-MHDA* 16-mercaptohexadecanoic acid; *PAN* poly(aniline); *CNTs* carbon nanotubes; *EDA* ethylenediamine; *PPIX* protoporphyrin IX; *TEOA* triethanolamine-functionalised; *MIP* molecularly imprinted polymer; *Fc-Lac* ferrocene lactose; *CG* carboxyl graphene; *MSN* mesoporous silica nanoparticles; *MB* methylene blue; *N-GNRs* N-doped graphene nanoribbons

An example of a sensor platform for BNP with an even lower LOD has been recently reported by Hu and co-workers [273]. They developed a novel enhanced photoelectrochemical platform based on the successive deposition of N-doped ZnO nanopolyhedra (N-ZnO NP) and protoporphyrin IX. The N-ZnO NP provided a low band gap of 2.6 eV and was utilised as the substrate to enhance the observed photocurrents. The sensing platform was produced through casting of protoporphyrin IX followed by the N-ZnO NP. The authors then cast a DNA aptamer onto the surface of the photoelectrochemical platform to produce an ultra-sensitive, label-free “signal-off” sensor. This exhibited a wide linear range from 1 pg mL^−1^ to 100 ng mL^−1^, with an LOD of 0.14 pg mL^−1^ and validation in human serum samples. In the majority of cases, the reported sensors provide very low detection levels. A thorough summary of commercial testing kits has recently been published for BNP [285] and inspection of Table 3 reveals that the detection levels are lower than commercial kits, indicating that they hold promise to be used in clinical settings. However, in some cases, the assay time can be longer than commercial kits, which is an area of future research focus.

#### N-terminal-pro hormone BNP (NT-proBNP)

**Fig. 7:**
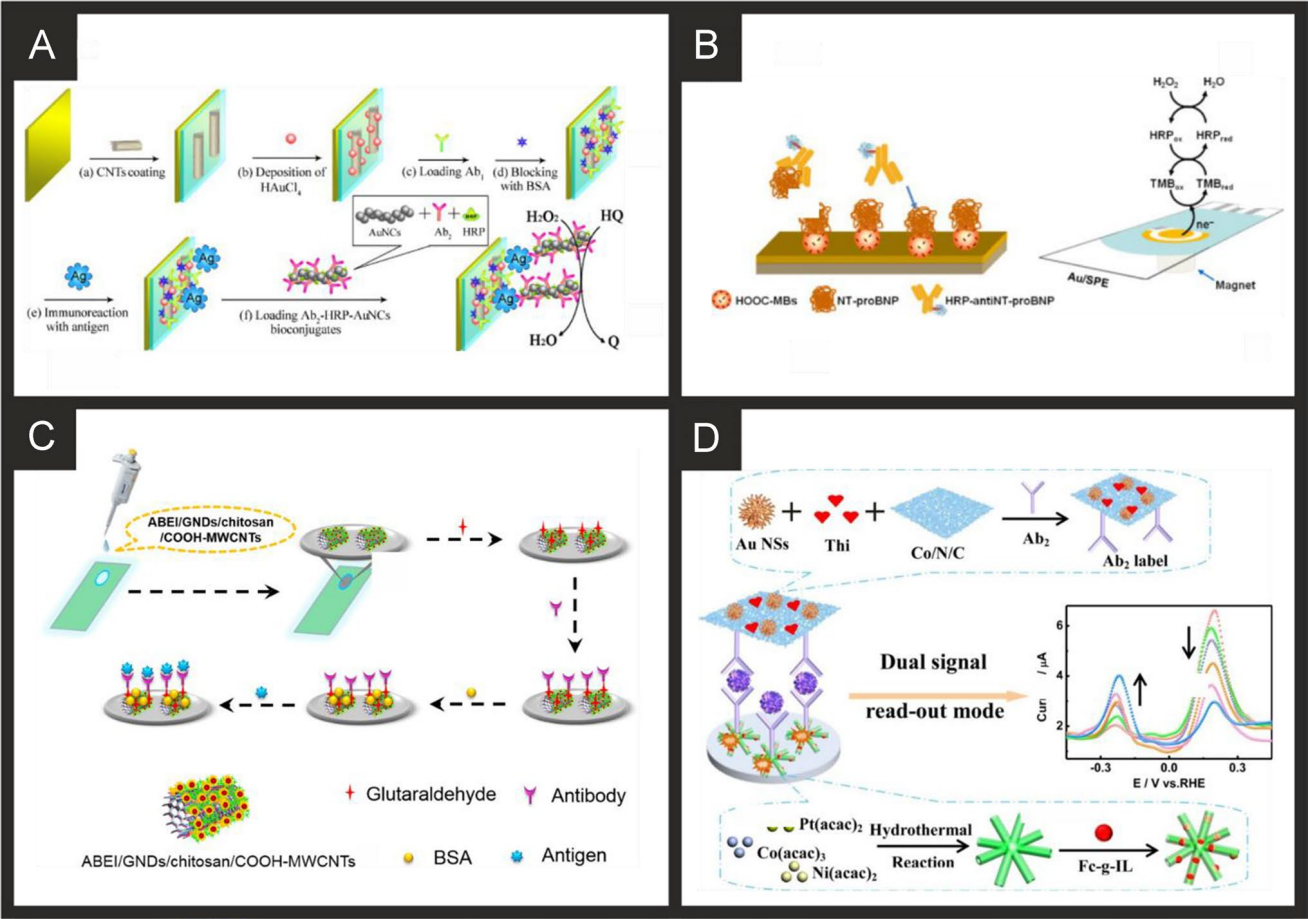
**A)** Schematic processes of the immunosensor fabrication based on Ab-HRP-AuNCs for the detection of NT-proBNP. Reproduced and adapted with permission from ref [241]. Copyright Elsevier 2011. **B)** Schematic showing the fabrication and detection methodology for the magnetoimmunosensor for the detection of NT-proBNP. Reproduced and adapted with permission from ref [243]. Copyright Elsevier 2013. **C)** Schematic description for the label-free NT-proBNP immunosensor based on ABEI/GNDs/chitosan/COOK-MWCNTs. Reproduced and adapted with permission from ref [247]. Copyright American Chemical Society 2015. **D)** Schematic of the production, composition and read-out of the ratiometric electrochemical immunoassay for NT-proBNP based on three dimensional PtCoNi hollow multi-branches/ferrocene-grafted-ionic liquid and Co–N-C nanosheets. Reproduced and adapted with permission from ref [261]. Copyright Elsevier 2021

The majority of sensors that have been developed to measure NT-proBNP are based on immunoassay with a limited amount using aptamer technology and none utilising the potential benefits of MIPs (see Table 3). Zhuo and co-workers [241] have reported an electrochemical sandwich immunosensor utilising a nano-structural gold and carbon nanotubes composite which provide immobilization sites for antibodies with gold nanochains and horseradish peroxidase (HRP) complex labelled secondary antibodies for signal amplification operating via a “signal on” mechanism; the sensor fabrication is shown in Fig. 7A. The signal amplification is based upon gold nanochains prepared by reducing a gold salt with ascorbic acid. The antibody was conjugated with the gold nanochains by simple stirring for 12 h, followed by centrifugation to remove excess reagents. HRP was then added to block the unmodified portion of the Au nanochains surface. The electrochemical platform comprises a gold macroelectrode onto which carbon nanotubes, synthesised by a chemical vapour deposition method, are immobilised. The nanotubes were acid treated to introduce carboxylic groups and then added into a solution containing a gold salt and electrochemically reduced to produce nanogold modified carbon nanotubes. Next, the addition of the antibody and BSA finishes the electrochemical platform. The immunosensor was shown to measure from 0.02 to 100 ng mL^−1^ with a LOD of 6 pg mL^−1^ using CV which would cover the ranges reported in the critically ill [63]. The sensor’s composition was reported by the authors to give rise to the sensitive sensing of NT-proBNP due to the carbon nanotubes promoting electron transfer and increasing the current response to hydrogen peroxide while the gold nanochains have more active sites than gold nanoparticles; thus, gold nanochains can immobilize more HRP and the current response to H_2_O_2_ is larger than that of the immunosensor using Au nanoparticles. No interference from cTnI, cTnT and cTnC were found, with the sensor found to be stable for up to 30 days. Esteban-Fernández de Ávila [243] have reported a novel amperometric magnetoimmunosensor using an indirect competitive format developed for the sensitive detection of NT-proBNP. Figure 7B shows a schematic representation of the sensor’s construction which involves the covalent immobilization of the antigen onto carboxyl-modified magnetic beads (HOOC-MBs) activated with EDC and sulfo-NHS, with further incubation in a solution containing variable concentrations of the antigen and a fixed concentration of an HRP-labelled detection antibody. Target NT-proBNP compete for binding with the specific HRP-labelled secondary antibody and the immunoconjugate-bearing MBs are captured by a magnet placed under the surface of a disposable gold SPE. Using amperometry, the analytical signal is measured using TMB (3,3′,5,5′-tetramethylbenzidine), an electrochemical mediator. The immunosensor measures NT-proBNP over the range 0.12 to 42.9 ng mL^−1^ with a LOD of 0.02 ng mL^−1^ and was shown to successfully measure NT-proBNP in human serum samples [243]. Such a dynamic range means the technology would prove useful for NT-proBNP sensing in the critically ill [63]. This study was extended to measure both NT-proBNP and CRP in human serum samples [286].

Zhang et al. [247] have developed an immunosensor based on a ITO electrode modified with carboxylic multi-walled carbon nanotubes, which have themselves been modified with chitosan, gold nanodots and N-(aminobutyl)-N-(ethylisoluminol). This is then modified with glutaraldehyde, immobilised through an amide reaction between the amino group of N-(aminobutyl)-N-(ethylisoluminol) and the aldehyde group of the glutaraldehyde. Last, the NT-proBNP antibody is connected via an amide reaction between the aldehyde group of glutaraldehyde and the amino group of the antibody. Figure 7C overviews the various steps of the sensor fabrication. Using ECL, the sensor was able to measure NT-proBNP over the range 0.01 to 100 pg mL^−1^ with a low LOD of 3.86 fg mL^−1^. The authors demonstrated the sensor selectivity by exploring the interferents cTnI, IgG, lysozyme, BSA which had little effect upon the sensors performance. The sensors performance was validated in human plasma samples and directly compared with ELISA giving comparable results, strongly suggesting that the developed sensor could be used for the quantitative analysis of NT-proBNP in real samples of human plasma. Such a dynamic range means the technology would prove useful for NT-proBNP sensing in the critically ill [63].

Chen and co-workers [261] have reported the development of a ratiometric immunosensor, the steps involved in its fabrication are shown in Fig. 7D. A GCE serves as the supporting electrode which is modified with PtCoNi hollow multi-branches nanostructures/ferrocene-grafted-ionic liquid. The PtCoNi hollow multi-branches nanostructures are fabricated via a hydrothermal method via mixing platinum, cobalt and nickel acetate salts with oleylamine and oleic acid under continuous ultrasonication followed but the addition of formaldehyde. The solution is then placed into an autoclave and reacted for 12 h at 190 degrees. Following this, they are washed and etched in acetic acid. The nanostructures are impressive and are composed of five or six hollow branches, with their length and width measured to be ~47 and 26 nm respectively. These PtCoNi hollow multi-branches nanostructures are drop cast onto a GCE after which the ferrocene-grafted-ionic liquid is also drop cast along with the antibody and last, BSA. The other part of the immunoassay is comprised of gold nanostars and Co–N-C nanosheets. The former is fabricated via a one-pot wet chemical reduction methodology where 5-hydroxymethyluracil is dissolved into water, adjusted to pH 10 with a gold salt added along with the reducing agent ascorbic acid, and stirred for 30 min. The final product was centrifuged and washed. The latter is obtained by a simple pyrolysis methodology. The gold nanostars, thionine and the Co–N-C nanosheets are mixed together in water, left overnight to incubate into a composite. The antibody is then added through dispersing this composite in phosphate buffer solution and leaving overnight. The immunosensor using DPV, which is a “signal on” sensor, was demonstrated to measure NT-proBNP over the range 0.001 to 10.0 ng mL^–1^ with a LOD of 0.35 pg mL^–1^, which again has limited use in the setting of HF diagnosis or prognostication in ICU. The selectivity of the sensor was explored towards possible interferents of cTnI, creatine kinase isoenzymes, neuron-specific enolase, and alpha fetoprotein which only caused very small changes in the peak currents towards the analytical target NT-proBNP (less than 5% RSD) and was able to successfully measure NT-proBNP in human serum. The authors attributed the sensors excellent analytical performance to be due to a combination of factors, enhanced electron transport and increased surface area from utilising 3D hollow PtCoNi multi-branches and improved biocompatibility from using the ferrocene-grafted-ionic liquid [287].

A common theme, as can be seen in Table 3, for the detection of this biomarker is the use of electrochemiluminescence (ECL). Li and co-workers [252] developed an ECL immunoassay based on the energy transfer from Luminol-Au@Fe_3_O_4_-Cu_3_(PO_4_)_2_ nanomaterials (ECL donor) to Au@CuS-rGO (ECL acceptor). In this approach, the former is immobilised upon a GCE which is then modified with Ab_1_ via incubation for 12 h, after which BSA is then immobilised upon rGO-Au@CuS-Ab_2_ to form the sandwich type immunoreaction mechanism. While the authors do not provide an exact mechanism, it is thought that it originated from ECL resonance energy transfer (ECL-RET) where the electrode materials promote electron transfer to luminol [252, 288]. The immunosensor was able to measure from 0.5 pg mL^−1^ to 20 ng mL^−1^ with a LOD of 0.12 pg mL^−1^. The authors validated their sensor in human serum and directly compared the results with ELISA which gave excellent agreement. In the above cases, and generally in biosensors, there is usually the incorporation of noble nanoparticles of various geometries and compositions, with generally the reason to increase electron transfer properties and provide binding sites. Indeed, at the nanoscale there are changes in the electronic structure and work has shown that reaction mechanism and kinetics differ at the nanoscale in comparison to the bulk [289]. The question of what type of nanoparticles provides the best electroanalytical sensor is a pertinent one. To this end, Beck et al. [259] explored a sandwich type assay for the detection of NT-proBNP, where a SPCE is modified with the capture Ab_1_ label (via drop casting) and silver and gold nanoparticle Ab_2_ labelled probes were explored and contrasted. The authors found that in both cases, NT-proBNP could be measured over the range 25 to 1000 ng mL^−1^ using DPV but found that through the use of silver nanoparticles, due to their greater electrochemical activity, they provide a six-times more sensitive assay [259]. The exploration of the geometry and composition of nanoparticles used in immunoassays should be a key future research direction.

### Neurohumoral markers

The detection of neurohumoral markers using electrochemical sensing platforms is sparce, with no examples found for the detection of MR-proADM or MR-proANP. This is an area that should be explored in future research. There have been some reports of electrochemical sensors for the detection of copeptin and that is where our attention turns next.

#### Copeptin

In terms of electrochemical based sensors, there are very few. Yang et al. [275] reported an ultrasensitive electrochemical immunoassay for copeptin determination using an ITO electrode modified with a RGO-TiO_2_ nanocomposite which is then in turn modified with Ab-copeptin/BSA via coupling with EDC/NHS; the TiO_2_ is in the form of nanoparticles prepared via a hydrothermal technique. The immunosensor using EIS was explored in model solutions where it was observed that as the concentration of copeptin rises, the capacitance declines, possibly because of variation in the dielectric/blocking traits of the electrolyte–electrode interface caused by the interaction between the antigen and antibody. Analysis of the EIS signal gives rise to a linear range of 10 to 500 ng mL^−1^ (249 to 12,344 pmol L^−1^) with a LOD of 0.15 ng mL^−1^ (37.3 pmol L^−1^). The interferent of ascorbic acid, uric acid, glucose, zinc, copper, mercury and cadmium salts were studied with no effect upon the electrochemical signal. The immunoassay has potential but clearly needs further work to demonstrate any potential clinical uptake. Han and co-workers [276] developed an ECL immunoassay for the measurement of copeptin based upon luminescence immune-gold nanoassemblies. The ECL immunosensor is based upon an ITO electrode onto which Cu^2+^—cysteine complexes and *N*-(aminobutyl)-*N*-(ethylisoluminol) functionalized gold nanoparticles combined with chitosan were drop cast and allowed to dry. Onto this modified ITO surface, gold nanoparticle labelled antibodies are fabricated via electrostatic interaction as well as hydrophobic interactions and weak covalent interactions [276]. This approach requires adding the copeptin antibody to a solution of prepared gold nanoparticles (12 nm diameter) followed by incubation overnight. Following this, BSA is added, and the conjugate centrifuged and cleaned with water. The antibody functionalised gold nanoparticles are simply drop cast onto the electrode surface and allowed to dry and the sensor is then ready to use. The sensor is a “signal off” where in the presence of copeptin, the ECL signal reduces due to the formation of antibody–antigen complexes. The ECL immunoassay exhibited a linear range from 0.02 to 10 pmol L^−1^ with a LOD of 0.0005 pmol L^−1^. The selectivity of the immunoassay was explored towards the interferents: Seven polypeptides and proteins including Y–H, 3Y–H, HGGG, MB, HAS, FABP and IgG, all at one order of magnitude higher than that of copeptin which indicated no detrimental effect upon the electrochemical signal. The authors went on to demonstrate the determination of copeptin in human serum with good recoveries (97.4–109.3%). Last, Qin et al. [277] reported the fabrication of a triethanolamine-functionalized Metallic Organic Framework (MOF) upon graphene oxide, both supported on a glassy carbon electrode which was then functionalised with anti-copeptin via modification with glutaraldehyde for 2 h after which the antibody was incubated via drop casting for 12 h. The next step was the addition of BSA and then, the sensor was ready to use via ECL Ru(bpy)_3_^2+^ redox probe. The linear range was from 5 pg mL^–1^ to 500 ng mL^–1^ (1.24 to 12,344 pmol L^−1^) with a LOD reported to correspond to 360 fg mL^–1^ (0.09 pmol L^−1^). The following interferents were explored upon the ECL signal GOx, human H-FABP, human cTnI, human IgG, l-cysteine, DA, and copeptin which were reported to have no effect; the authors demonstrated the successful determination of copetin in human serum with good recoveries (96–104%). This target has scope for future develop based on aptamer and MIP technology.

### Markers of extracellular matrix remodelling

As with the previous section, there is very little published literature on the electrochemical detection of the markers of extracellular matrix remodelling. None were found for GDF-8 or GDF-15, and we suggest this to be a productive area of future research. There were some examples for GAL-3 and sST2 and that is where our attention turns first.

#### Soluble suppression of tumorigenicity 2 (sST2)

There is limited literature on the electrochemical detection of sST2, see Table 3, with some alternative detection methods explored [290]. Demirbakan and Sezgintürk reported a sST2 immunoassay using disposable graphite paper (GP) electrodes [279]. The authors took inspiration from the battery field where paper electrodes are common and have reported advantages which include: very low-cost, high electrical conductivity and practical immobilization methods. The GP utilised in this work is 0.3 mm thick with a size of 210 mm × 210 mm and was commercially purchased. The GP was modified with C_60_ via drop-casting before incorporating carboxyl groups through the application of sulfuric acid. Following this they immobilised anti-sST2 with EDC/NHS and blocked the remaining surface with BSA. Using EIS they reported a linear range from 0.1 to 100 fg mL^−1^, with a LOD of fg mL^−1^ and show that the sensor platform can be stored for 10 days at 4 ºC whilst only losing 4.48% of the performance. The sensor shows no interference from cysteine, heat shock protein, protein activated kinase 2 or TNF-α and was validated through the detection in human serum samples, achieving recoveries between 100 and 113.46%.

Recently, Torrente-Rodríguez and co-workers described an electrochemical sandwich immunoassay for sST2 using SPEs [278]. They developed magnetic immunoconjugates through EDC/NHS coupling of capture antibodies onto the surface of commercially procured carboxylic acid-modified magnetic beads. Upon the binding of target sST2 and secondary antibody labelled with streptavidin and HRP was introduced to allow for a measurable signal using chronoamperometry which occurs via a “signal on” mechanism. This system required an incubation time of sST2 of 15 min, followed by an incubation time of 30 min for the secondary labelled antibodies. Using this methodology, they achieved a linear range between 76 and 2500 pg mL^−1^, based on loading of 50 µg mL^−1^ of capture antibody, and a LOD of 26.7 pg mL^−1^. The sensor was used to detect the presence of sST2 in 25-times diluted human plasma samples from healthy individuals, which exhibited no matrix effects. The results from this were validated against a commercial ELISA platform, where no statistically significant differences were observed. Although the approaches to sST2 detection described in the literature have achieved excellent sensitivities, efforts now need to focus on refining the technology for optimal detection within physiologically relevant concentrations [108, 111].

#### Galectin-3 (Gal-3)

There have been a few recent reports on the development of electrochemical biosensors for the detection of Gal-3. The first by Tang and co-workers reported a sandwich type immunosensor that utilised various materials to enhance the sensor performance such as metal–organic frameworks (MOFs), AuNPs and nitrogen-doped graphene nanoribbons (N-GNRs) [283]. The GCE was modified with N-GNRs-Fe-MOFs@AuNPs; firstly, the N-GNRs were produced through mixing nitrogen-doped MWCNTs with H_2_SO_4_ and H_3_PO_4_ at 140 ºC followed by the addition of KMnO_4_ at 65 ºC. The Fe-MOFs were produced separately [291] and decorated with AuNPs through reduction with NaBH_4_. The N-GNRs and Fe-MOFs@AuNPs were then combined through sonication and stirring then drop-cast onto the surface of the GCE, before being modified with the Gal-3 specific antibody and the remaining active surface blocked with BSA. The other half of the sandwich was a AuPt-methylene blue (AuPt-MB) nanocomposite whereby, the methylene blue was allowed to form micelles in a solution of HCl and dodecyltrimethylammonium bromide (DTAB), followed by the addition of HAuCl_4_ and H_2_PtCl_6_ to form the nanoparticles. The secondary antibody was incubated with these nanocomposites for 12 h at 4 ºC before further blocking with BSA to prevent non-specific adsorption. For the detection of Gal-3 a 6 µL sample was incubated onto the modified GCE surface for 1 h at 37 ºC, before incubation with the AuPt-MB-Ab_2_ nanocomposite for a further 1 h at 37 ºC. Using DPV they reported a linear relationship for the detection of Gal-3 from 100 fg mL^−1^ to 50 ng mL^−1^, achieving a LOD of 33.33 fg mL^−1^. They attribute the performance of the platform to the synergistic effect of the N-GNRs-Fe-MOFs@AuNP and AuPt-MB, with the former providing good electrical conductivity and a larger electroactive surface area and the latter providing good biocompatibility, high loading of antibodies and good signal amplification. They tested the sensing platform in human serum achieving recoveries between 97.99 and 104.84%, further validating it against a commercial ELISA platform showing the sensor provided satisfying accuracy. Although promising, as the authors note, the production time of the sensor is too long for commercial use. Additionally, a cost analysis of the sensor would be useful as, there is a large number of different materials and the use of a GCE, which could be problematic for the transition to clinical care.

Another sandwich immunoassay utilising methylene blue has been reported by Liu et al*.* [282]. They created their capture platform through drop-casting carboxyl graphene (CR) onto the surface of a GCE before electrochemically reducing it, conjugating the capture antibody through EDC/NHS coupling and finally, blocking the remaining surface with BSA. The secondary antibodies were attached to mesoporous silica nanoparticles along with AuNPs and MB. They utilise both the MB and AuNPs for the detection through using DPV to monitor the reduction of MB and ASV for the oxidation of the AuNPs. The detection using the Au required the use of *aqua regia* to give a well-defined anodic Au-stripping peak; however, it is difficult to see how this would be used in a clinical setting. Using the MB detection method, they achieved a linear range from 50 fg mL^−1^ to 500 ng mL^−1^ and a LOD of 2 fg mL^−1^. They showed that the immunosensor production was reproducible over a batch of 30 electrodes, giving a RSD of 6.4%. Additionally, the sensor exhibited minimal interference from the presence of various other proteins and was shown to work in clinical serum samples, with the MB sensing method producing recoveries between 95.8 and 106%.

Piguillem and co-workers described a sandwich assay for Gal-3 based on the use of commercially procured carboxylic acid modified magnetic beads (MBs), allowing for the electrochemical measurements to be performed in buffer solution rather than the more difficult blood samples [281]. Capture antibodies were conjugated to carboxyl modified MBs through EDC/NHS coupling followed by blocking with ethanolamine. These Ab-MBs were then incubated in a solution containing Gal-3, followed by further incubation with a detection Ab and then streptavidin-HRP. After all the incubations, the solutions were washed and stored in phosphate buffer, in which the amperometric measurements could be performed. For the measurements, this solution was dropped onto an SPCE surface and inserted into a solution of 1 mM hydroquinone, followed by the addition of H_2_O_2_ and amperometric detection at -0.2 V (*vs.* Ag *pseudo*-reference). Through this methodology, they achieved a linear detection range between 0.028 to 5 ng mL^−1^ and a LOD of 8.3 pg mL^−1^. The production methodology had a RSD of 7.7% and was validated in clinical plasma samples from both health individuals and those that had experienced heart failure against commercial ELISAs. The authors continued to show the multiplex possibilities of this sensing platform, using a dual SPCE, for the detection of both Gal-3 and NT-proBNP in buffered samples.

Finally, Cerqueira et al. [284] have recently reported a MIP based biosensor for the detection of Gal-3. The MIPS were formed onto the surface of a SPCE using CV electropolymerisation of the aminophenol monomer in the presence of Gal-3 protein (5 µg mL^−1^). The template was removed through incubation of a solution of oxalic acid (0.5 M) overnight, before thoroughly washing and storage in PBS. Detection of Gal-3 was achieved using EIS, with a dynamic range from 0.005 to 50 µg mL^−1^. They showed that the sensor was capable of detecting Gal-3 in human serum samples, reporting an LOD 10 times lower than in buffer which is not explained. This sensor shows a glimpse of what can be achieved with MIPs for the development of these sensor platforms, and we expect further work to be published in this area.

### Inflammatory markers

There are a significant number of reports of the development of electrochemical biosensors for inflammatory biomarkers due to the wide range of uses for them throughout healthcare, and not just as markers of cardiac dysfunction, see Table 4. As such we will focus on the last 5 years for each marker discussed beginning with IL-6.

**Table 4:** A summary of the reported literature for the electrochemical detection of the inflammatory markers linked to cardiac disease; highlighting the marker(s) targeted, electrode materials and modifications, and the electroanalytical method used alongside the measured linear range, limit of detection and real sample medium

Cardiac biomarker	Electrode material	Sensor composition	Electroanalytical method	Dynamic range	Limit of detection	Real sample	Reference
IL-6	Gold	MPA/Ab_1_/StreptavidinMBs/HRP/Ab_2_	DPV	0.05–5000 pg mL^−1^	0.05 pg mL^−1^	Human Serum	[292]
IL-6	GCE	r-GO/Fe_3_O_4_/PDDA/CdSe/Ab	ECL	0.002–20 ng ml^−1^	0.65 pg mL^−1^	Human Serum	[293]
IL-6	Gold	Diazonium/GO/PCC/Ab_1_/Ab_2_/GO/NB	SWV	1–300 pg mL^−1^	1 pg mL^−1^	Cell Culture	[294]
IL-6	GCE	Pt–Pd NPs/Ab	LSV	0.1–200 pg mL^−1^	0.032 pg mL^−1^	Human Serum	[295]
IL-6	GCE	Ru(bpy)_3_^2+^@AMCs/Ab_1_/Ab_2_-HRP/ACP/OAMs	ECL DPV	10^–5^-9000 pg mL^−1^ 10^–3^-9000 pg mL^−1^	3.5 × 10^–6^ pg mL^−1^ 3.2 × 10^–4^ pg mL^−1^	Human Serum	[296]
IL-6 TNF-α	GCE	4-AB/PPC/Ab_1_/Ab_2_/GO/Fc OR MtB	SWV	5–150 pg mL^−1^ 5–200 pg mL^−1^	5 pg mL^−1^ 5 pg mL^−1^	Mouse Serum	[297]
IL-6	FTO	LaFeO_3_/chitosan/Ab	Photoelectrochem	0.1 pg mL^−1^–0.1 µg mL^−1^	33 fg mL^−1^	Human Serum	[298]
IL-6	Gold microelectrode	Sulfo-LC-SPDP/DTT/Ab	DPV	0–60 pg mL^−1^	20 pg mL^−1^	Human Serum	[299]
IL-6 TNF-α	Gold SPE	SAM/Ab_1_/Ab_2_/HRP	Amperometry	-	8 ng mL^−1^ 2 ng mL^−1^	Differentiation Medium	[300]
IL-6	Gold	BSA/AuNW/GA/Ab_1_/Ab_2_/streptavidin/HRP	CV	~ 5–500 pg mL^−1^	4 pg mL^−1^	Human Plasma	[301]
IL-6	GCE	4-AB/ATP/AuNPs/Aptamer	EIS	5–100000 pg mL^−1^	1.6 pg mL^−1^	Human Serum	[302]
IL-6	ITO	PPyr-NHS/Ab	EIS	0.03–22.5 pg mL^−1^	10.2 fg mL^−1^	Human Serum	[303]
IL-6	GCE	CG/Ab_1_/Ab_2_/NiCoO_2_@CeO_2_ NBs	Amperometry	2.5 × 10^–5^-10 ng mL^−1^	7 fg mL^−1^	Human Serum	[304]
IL-6	ITO	PPCE/IL 6R	EIS	0.02–16 pg mL^−1^	6 fg mL^−1^	Human Serum	[305]
IL-6	ITO	AcB/EpxS-PPyr/IL 6R	EIS	0.01–50 pg mL^−1^	3.2 fg mL^−1^	Human Serum	[306]
IL-6	SPCE	PPy-MIP	EIS	0.02–20000 pg mL^−1^	0.1 pg mL^−1^	Human Serum	[307]
IL-6	ITO-PET	DHBA-TiO_2_/Ab	Photoelectrochem	2–2000 pg mL^−1^	3.6 pg mL^−1^	Human Plasma	[308]
IL-6	Gold	ZnO/Ab	EIS	0.01–10000 pg mL^−1^	0.1 pg mL^−1^	Human Plasma	[309]
IL-6	Gold	MPA/Ab	DPV	1 pg mL^−1^–1 µg mL^−1^	1.63 pg mL^−1^	Human Serum	[310]
IL-6	GCE	NMC@AuNP/Ab	DPV	0.5–1200 pg mL^−1^	0.14 pg mL^−1^	Human Serum	[311]
IL-6	ITO	Bi_2_S_3_/Bi_2_MoO_6_/Ab/SiO_2_/alkaline phosphatase	Photoelectrochem	50 fg mL^−1^–10 ng mL^−1^	20 fg mL^−1^	Human Serum	[312]
CRP	ITO	rGO/AuNP/MPA/Ab	EIS	1–10,000 ng mL^−1^	0.08 ng mL^−1^	Human Serum	[313]
CRP	Gold	ssDNA/Ab	EIS	3.125–25 mg L	-	Human Serum	[314]
CRP	GCE	GQD/Ab	EIS	60–8400 ng mL^−1^	21.12 ng mL^−1^	Human Serum	[315]
CRP	SPCE	rGO/PyNHS/Ab	EIS	10 ng mL^−1^–10 µg mL^−1^	-	-	[316]
CRP	Au-SPE	Ab/BSA	DPV	6.25–50 µg mL^−1^	0.78 µg mL^−1^	Negative Serum	[317]
CRP	Gold	DNA/thiolated-aptamer	SWV	120–12000 ng mL^−1^	120 ng mL^−1^	Human Serum	[318]
CRP	GCE	PEI-Fc/Ab	DPV	1–5 × 10^4^ ng mL^−1^	0.5 ng mL^−1^	Rat Blood	[319]
CRP	ITO	CUTMS/PAMAM/Ab	EIS	21–6148 fg mL^−1^	0.34 fg mL^−1^	Human Serum	[320]
CRP	CPE	IL/MPC/ZnO/Ab	DPV	0.01–1000 ng mL^−1^	5 pg mL^−1^	Human Serum	[321]
CRP	ITO	Nafion/Pt-NWs/TiNTs/Ab	ECL	0.05–6.25 ng	0.011 ng	Human Serum	[322]
CRP	Gold	MPA/Ab/BSA	SWV	5–220 fg mL^−1^	2.25 fg mL^−1^	Human Serum	[323]
CRP	SPCE	CDP-choline/chitosan	EIS	0.005–500 mg L^−1^	0.001 mg L^−1^	Human Serum	[324]
CRP	SPCE	AuNPs/PMPC-SH	DPV	5–5000 ng mL^−1^	1.6 ng mL^−1^	Human Serum	[325]
CRP	SPCE	AuNPs/L-cysteine/Ab	EIS	0.05–100 µg mL^−1^	15 ng mL^−1^	Human Serum	[326]
CRP	GCE	Bacteriphage/CNF	CV	0.04–100 µg mL^−1^	0.04 µg mL^−1^	Human Serum	[327]
CRP	GE	rGO/polytyramine/Ab	DPV	1.09–100 µg mL^−1^	1.25 µg mL^−1^	Human Serum	[328]
CRP	GCE	MBs/Ab_1_/Ab_2_/Ir-dmpq	ECL	0–600 ng mL^−1^	1 ng mL^−1^	ProCell Solution	[329]
CRP	CF	Bent-MWCNT/Ab	EIS	10–100 ng mL^−1^	4.8 ng mL^−1^	Human Whole Blood	[330]
CRP	GN-SPE	PANI/phytic acid/Ab	EIS	0.25–2 µg mL^−1^	0.5 µg mL^−1^	Fetal Bovine Serum	[331]
CRP	SPCE	MBs/streptavidin/Ab_1_/Ab_2_/HRP	Amperometry	0.005–1 µg mL^−1^	1.5 ng mL^−1^	Human Whole Blood	[332]
CRP	SPCE	Streptavidin/rGO/Ni/PtNPs/Ab_1_/Ab_2_/HRP	Amperometry	2–100 µg mL^−1^	0.8 µg mL^−1^	Preterm Baby Plasma	[333]
CRP	GCE	Chitosan/AuNPs/IL/MoS_2_/Ab_1_/Ab_2_/IrNPs/GO-DN	Amperometry	0.01–100 ng mL^−1^	3.3 pg mL^−1^	Human Serum	[334]
CRP	Gold	Streptavidin/rGO/Ni/PtNPs/Ab_1_/Ab_2_/HRP	Amperometry	1–100 µg mL^−1^	0.54 µg mL^−1^	Preterm Baby Plasma	[335]
CRP	Gold	Peptide/Ab	EIS	60–1200 ng mL^−1^	28.8 ng mL^−1^	-	[336]
CRP	SPCE	AuNPs/Ab	Amperometry	1–100 µg mL^−1^	0.085 µg mL^−1^	Human Serum	[337]
CRP	SPCE	GO/Ab	SWV	0.001–100 µg mL^−1^	0.38 ng mL^−1^	Human Serum	[338]
CRP	SPCE	AuNPs/MEL/Fc-ECG	DPV	0.001–1000 µg mL^−1^	0.30 ng mL^−1^	Human Serum	[339]
CRP	GCE	PTB7-Th/AuNPs/aptamer	Photoelectrochem	0.12–120000 ng mL^−1^	0.0396 ng mL^−1^	Human Serum	[340]
CRP	Gold	MBA/APBA/Ab/glucose	EIS	10–100 ng mL^−1^	1.2 ng mL^−1^	Calf Serum	[341]
CRP	Gold	Ferrocenethiol/phenylalanine/Ab	CV	1.2–1200 ng mL^−1^	0.192 ng mL^−1^	Human Serum	[342]
TNF-α	ITO	PPC-PBA/Ab_1_/Ab_2_/HRP	Amperometry	0.01–500 ng mL^−1^	10 pg mL^−1^	Whole Blood	[343]
TNF-α	SPCE	Au-graphene/chitosan/Aptamer/Ag@Pt	DPV	5–70 pg mL^−1^	1.64 pg mL^−1^	Human Serum	[344]
TNF-α	SPCE	MBs/affibody/Ab/alkaline phosphatase	DPV	76–5000 pg mL^−1^	38 pg mL^−1^	Human Serum	[345]
TNF-α	Gold	rGO/AuN/PPC/Ab_1_/Ab_2_/GO/Fc	SWV	0.1–150 pg mL^−1^	0.1 pg mL^−1^	Live Cells	[346]
TNF-α	SPCE	HOOC-Phe-DWCNTs/Ab_1_/Ab_2_/Streptavidin/HRP	Amperometry	1–200 pg mL^−1^	0.85 pg mL^−1^	Human Serum & Saliva	[347]
TNF-α	FTO	TiO_2_-NAs/CdS:Mn^2+^/Ab	Photoelectrochem	0.002–200 ng mL^−1^	1 pg mL^−1^	Human Serum	[348]
TNF-α	ITO	Ab_1_/Ab_2_/MB/CdS	ECL	1.6–200 pg mL^−1^	1.6 pg mL^−1^	Human Serum	[349]
TNF-α	Gold	PMMA/FNAB/Ab_1_/Ab_2_/streptavidin/alkaline phosphatase	DPV	0.1–100 ng mL^−1^	112.1 pg mL^−1^	Human Serum	[350]
TNF-α	Gold	DTSP/Ab_1_/Ab_2_/alkaline phosphatase	DPV	0.5–100 ng mL^−1^	60 pg mL^−1^	Human Serum	[351]
TNF-α	GCE	Fe_3_O_4_@AuNP/Aptamer	SWV	0.01–100 ng mL^−1^	10 pg mL^−1^	Human Serum	[352]
TNF-α	Gold	CMA/Ab	EIS	13–666 ng mL^−1^	-	-	[353]
TNF-α	ITO	CMA/Ab	EIS	10–100 pg mL^−1^	5 pg mL^−1^	-	[354]
TNF-α	Gold	CMA/Ab_1_/Ab_2_/HRP	Amperometry	1–30 pg mL^−1^	1 pg mL^−1^	Human Saliva	[355]
TNF-α	GCE	Cr-AuNCs/MnO_2_	ECL	0.06–31 pg mL^−1^	36 fg mL^−1^	Human Serum	[356]
TNF-α	GCE	AuNPs/aptamer_1_/aptamer_2_/Ru(phen)_3_^2+^/GO	ECL	0.005–5 ng mL^−1^	0.1 ng mL^−1^	Cell Secretion	[357]
TNF-α	SPCE	Neu-MBs/Ab_1_/Ab_2_/HRP	Amperometry	16–1000 pg mL^−1^	3 pg mL^−1^	Human Serum	[358]
TNF-α	GCE	CeNF/Nafion/Ab	EIS	10 fg mL^−1^–1 ng mL^−1^	1.2 fg mL^−1^	Human Plasma	[359]
TNF-α	ITO	ZIF-8@ZnO/MQDs/aptamer/MB	DPV	10 fg mL^−1^–0.5 µg mL^−1^	6.14 fg mL^−1^	Human Serum	[360]
TNF-α	ITO	CD-PMMA/Ab	Amperometry	0.05–160 pg mL^−1^	1.39 pg mL^−1^	Human Serum	[361]

*IL-6* interleukin-6; *GCE* glassy carbon electrode; *MPA* mercaptopropionic acid; *Ab* antibody; *MBs* magnetic beads; *HRP* horseradish peroxidase; *DPV* differential pulse voltammetry; *r-GO* reduced graphene oxide; *PDDA* poly(diallyl-dimethylammonium chloride); *ECL* electrochemiluminescence; *GO* graphene oxide; *PCC* 4-aminohenyl phosphorylcholine; *NB* nile blue; *SWV* square-wave voltammetry; *NPs* nanoparticles; *LSV* linear sweep voltammetry; *AMCs* anatase mesocages; *ACP* acid phosphatase; *OAMs* octahedral anatase mesocrystals; *4-AB* 4-aminobenzoic acid; *PPC* 4-aminophenyl phosphorylcholine; *Fc* ferrocene; *MtB* methylene blue; *FTO* fluorine-doped tin oxide; *Sulfo-LC-SPDP* sulfosuccinimidyl 6-[3’-(2-pyridyldithio) propionamido] hexanoate; *DTT* dithiothreitol; *SAM* self-assembled monolayer; *CV* cyclic voltammetry; *ATP* aminothiophenol; *ITO* indium-doped tin oxide; *PPyr-NHS* N-succinimidyl ester polypyrrole; CG: carboxylated graphene; *NBs* nanoboxes; *PPCE* conjugated polypyrrole with epoxy active side groups; *IL 6R* interleukin-6 receptor; *EpxS-PPyr; AcB* acetylene black; *EpxS-PPyr* epoxy substituted-polypyrrole polymer; *PPy-MIP* polypyrrole; molecularly imprinted polymer; *PET*: poly(ethylene terephthalate); *DHBA* 3,4-dihydroxybenzaldehyde; *MPA* mercaptopropionic acid; *NMC* nanoporous mesoporous carbon; *GQD* graphene quantum dot; *PyNHS* 1-pyrenebutyric acid *N*-hydroxy succinimide ester; *PEI* polyethyleneimine; *CUTMS* 11-syanoundecyltrimethoxysilane; *PAMAM* polyamidoamine; *CPE* carbon paste electrode; *IL* ionic liquid; *MPC* mesoporous carbon matrix; *NWs NTs* nanotubes; *PMPC-SH* thiol-terminated poly(2-methacryloyloxyethyl phosphorylcholine); *CNF* carbon nanofibers; *GE* graphite electrode; *Ir-dmpq* iridium (III) acetonitrile complex with 2-(3,5-dimethylphenyl)quinoline; *CF* carbon film; *GN-SPE* graphene nanoplatelet screen printed electrode nanowires; *GO-DN* graphene oxide-1,5-diaminophthalene; *MEL* melamine; *Fc-ECG* ferrocene modified reduced glutathione; *PTB7-Th* poly(4,8-bis[5-(2-ethylhexyl) thiophen-2-yl] benzo[1,2-b:4,5-b’]dithiophene-2,6-diyl-alt-3-fluoro-2-[(2-ethylhexyl)carbonyl] thieno[3,4-b]-thiophene-4,6-diyl); *MBA* 4-mercaptobenzoic acid; *APBA* 4-aminophenylboronic acid; *PCC-PBA* phenyl phosphorylcholine–phenyl butyric acid; *NAs* nanorod arrays; *FNAB* 4-fluoro-3-nitro-azidobenzene; *PMMA* polymethyl methacrylate; *DTSP* dithiobis(succinimidyl proponate); *CMA* 4-carboxymethyl aryl diazonium; *Neu-MBs* neutravidin functionalised magnetic beads; *CeNF* cerium oxide nanofibers

#### Interleukin-6 (IL-6)

From inspection of Table 4, the majority of proposed electrochemical sensors for IL-6 are immunoassay based with some examples of aptamer and MIP based platforms. Tang and co-workers [292] reported a microfluidic immunoassay for the multiplexed detection of cancer biomarkers, including IL-6. In this work, they produced a 32-sensor array (8 electrodes per biomarker analysed) using gold electrodes modified first with a mercaptopropionic acid (MPA) self-assembled monolayer (SAM), followed by EDC/NHS coupling of the specific primary antibodies. The secondary antibodies and HRP tag were chemically attached to streptavidin-modified magnetic nanoparticles (300 nm) and drawn by a syringe into the fluidic chambers. Detection, operating via a “signal on” approach was achieved through the injection of hydroquinone and hydrogen peroxide into the microfluidic detector channels using individual syringes, with the resulting DV measurements staggered to account for the time delay in the multiplexer. Through the use of the multiplexer, they managed to connect 8 of their microfluidic devices together as one, allowing for the analysis of 256 sensors in a time of 30 min. The authors validated their results with internal controls by having 2 of every 8 sensors incorporate BSA instead of antibodies and then further validation through comparison to ELISAs. Examples such as this providing a large throughput of samples show promise for further development towards ICU settings.

**Fig. 8:**
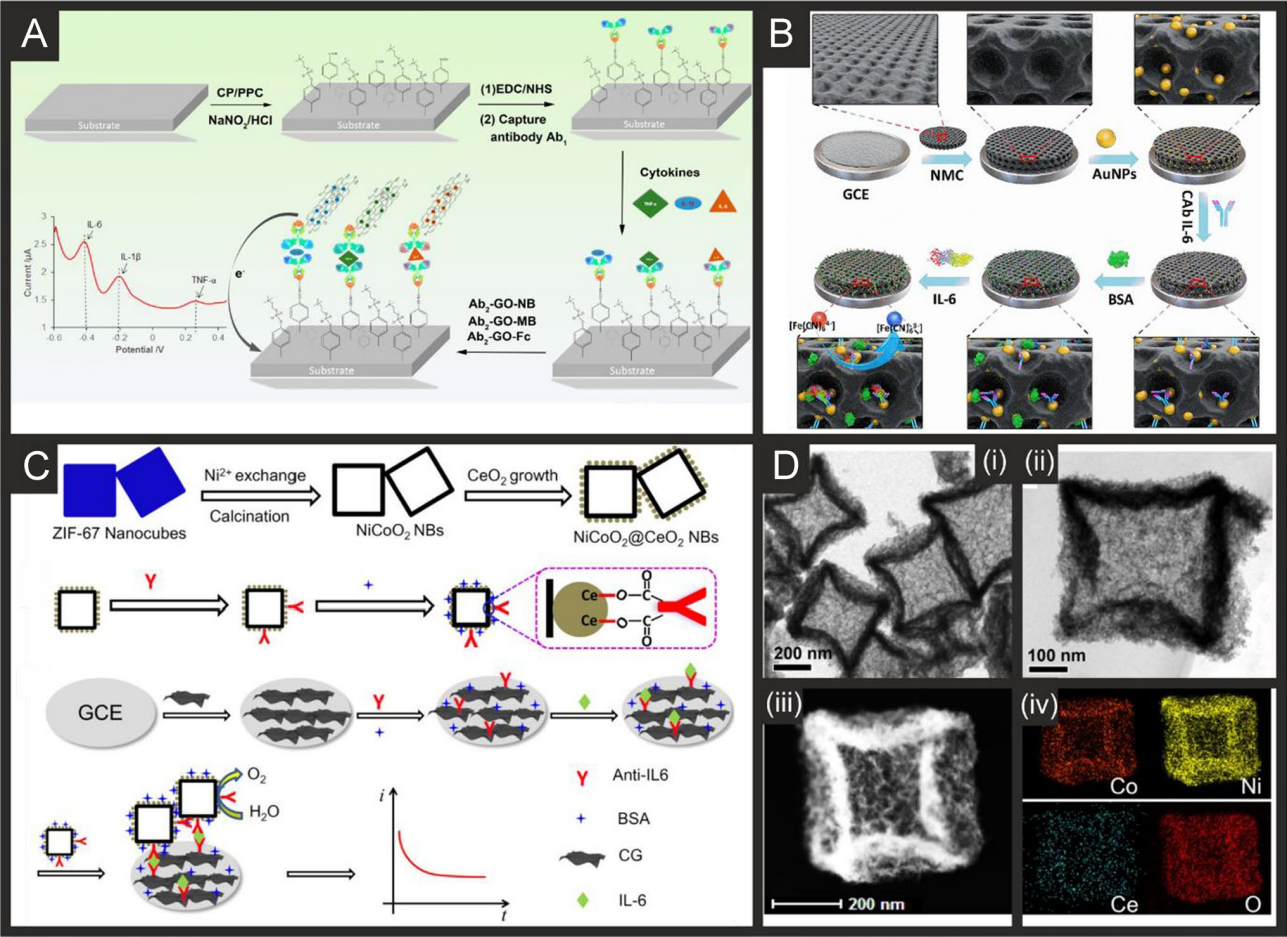
**A)** Schematic of the sensor fabrication and representative DPV for the simultaneous immunosensing of multiple cytokines in serum. Reproduced and adapted with permission from ref [297]. Copyright American Chemical Society 2018. **B)** Schematic illustration of the immunosensor production and working mechanism based on porous carbon composites. Reproduced and adapted with permission from ref [311]. Copyright Elsevier 2021. **C)** Illustration of the synthesis procedure for NiCoO_2_@CeO_2_ NBs, the preparation of the electrocatalytic labels and the fabrication of the immunosensor. Reproduced and adapted with permission from reference [304]. Copyright American Chemical Society 2020. **D)** (i,ii) TEM images of NiCoO_2_@CeO_2_ NBs; (iii,iv) STEM image and elemental mapping of NiCoO_2_@CeO_2_ NBs. Reproduced and adapted with permission from ref [304]. Copyright American Chemical Society 2020

An alternative multi-marker approach is reported by Wei et al. [297] for the simultaneous detection of IL-6, IL-1β and TNF-α. They achieve this through the immobilisation of specific capture antibodies for the three analytes targeted onto the GCE surface through diazonium salt electrodeposition followed by EDC/NHS coupling (Fig. 8A). When the target has bound to the surface an incubation of the secondary antibody solution is performed for 30 min. This solution contained specific antibodies for the three targets, each tagged with a different redox probe; Nile blue (NB, -0.4 V) for IL-6, Methylene blue (MB, -0.2 V) for IL-1β and Ferrocene (Fc, + 0.2 V) for TNF-α. This allowed for a DPV signal to be obtained for each individual biomarker based on the appearance of the oxidation peak corresponding to that specific redox tag (Fig. 8A (left)). Through this methodology, IL-6 was able to be detected in the range of 5 to 150 pg mL^−1^ with a detection limit of 5 pg mL^−1^. There was no significant interference observed from the presence of BSA, IgG PSA and CA-125 and the results were further validated in mouse serum. This system doesn’t meet the LOD requirements for identification of IL-6 in healthy individuals but could be used to indicate elevated levels in heart failure. From an ICU standpoint, the availability of such as a multi-senor would not only be useful for stratification in conditions such as HF and AMI, but would also be useful in the monitoring of other critical illnesses such as sepsis and COVID-19, provided its dynamic range was extended to accommodate those concentrations in excess of 50 ng mL^−1^ seen in septic patients [94].

Liu and co-workers [296] have reported a dual-responsive sandwich immunoassay that utilises both electrochemical and ECL for the detection of IL-6. They modified a GCE surface with a composite of TiO_2_ anatase mesocages (AMCs) and a carboxy-terminated ionic liquid (IL), followed by the ECL probe Ru(bpy)_3_(II) and their IL-6 specific capture antibody. The AMCs and IL are utilised for immobilisation of high loadings of the other components. The mesocages were prepared by taking sodium dodecyl sulfate dissolved in hydrochloric acid solution to which titanium (IV) isopropoxide was added and kept at 80 °C for 48 h under stirring; final products were obtained by centrifugation washed thoroughly with distilled water and dried at 60 °C overnight, and then calcined at 400 °C for 30 min in air to remove the residual organics. The second part of the sandwich assay is comprised of octahedral anatase mesocrystals (OAMs), functionalised with acid phosphatase (ACP), and HRP labelled secondary antibodies. The OAMs were synthesised by taking titanate nanowires dispersed in acetic acid and then transferred into a Teflon-lined stainless steel autoclave at 200 ºC for 48 h. The resulting precipitated was obtained by centrifugation and washed with distilled water and ethanol. The final product was attained by drying the precipitate at 60 ºC for 12 h and calcined at 400 ºC for 30 min to remove the residual organics. The OAM has high crystallinity, photoelectric activity, and a nano-porous structure for immobilisation. Using the electrochemical sensing methodology, they achieved a linear range between 10 fg mL^−1^ and 90 ng mL^−1^, with a LOD of 0.32 fg mL^−1^; whereas using ECL they achieved a linear range between 10 ag mL^−1^ to 90 ng mL^−1^ and a LOD of 3.5 ag mL^−1^. This was tested in human serum with good recoveries. This platform showed promise, however a new ECL probe would be required for commercialisation due to possible leaking of the Ru(bpy)_3_(II) from solid state biosensors.

A non-sandwich immunoassay based system with an appropriate wide linear range (0–1200 pg mL^−1^) and low LOD (0.14 pg mL^−1^) was reported by Liu et al. [311] (Fig. 8B). They utilised a hierarchical nanoporous mesoporous-carbon composite (NMC) decorated with AuNPs on a GCE as the base for their platform. The NMC was formed through SiO_2_-nanoparticle assisted sacrificial strategy, achieving an interconnected 3D network with high surface area for deposition of the AuNPs. The anti-IL-6 was then conjugated to the AuNPs through EDC/NHS coupling before blocking the remaining active surface with BSA. DPV was used for the detection of IL-6, with the proposed platform validated in human serum against commercially available ELISA kits producing recoveries from 82.1 to 117%. Cao and co-workers [304] used nanocubes as a high surface area component for loading of their secondary antibodies and as a detection element (Fig. 8C). They used Ni^2+^ exchange, calcination and the CeO_2_ growth to produce NiCoO_2_@CeO_2_ nanoboxes from ZIF-67 (a cobalt-based zeolitic imidazolate framework) (Fig. 8D). These nanoboxes exhibit a catalytic effect toward the oxygen evolution reaction, which changed upon binding of the target analyte. The CeO_2_ nanoparticles served a dual purpose of enhancing the catalytic effect and providing sites for facile surface immobilisation of the antibodies through ester-like bridging. The GCE surface itself was modified with carboxylated graphene followed by EDC/NHS coupling of anti-IL-6 and blocking with BSA. For detection, sample was incubated onto the electrode for 50 min followed by the secondary antibody and nanocube system for 50 min. OER testing at + 1.3 V (*vs.* SCE) was used for detection, measuring the amperometric response, achieving a linear range from 2.5 × 10^–5^ to 10 ng mL^−1^ and a LOD of 7 fg mL^−1^. The authors showed that this platform performed well (93.8%) for up to 30 days post fabrication and validated their results in human serum samples against a commercial ELISA kit, achieving recoveries between 95.5 and 104%. For this system to be suitable for commercial uptake the two-step incubation times would need to be reduced from the current 1 h 40 min.

Last, Tanak and co-workers have reported a multiplex system for cytokine detection, including IL-6, IL-8, IL-10, TRAIL and IP-10 in undiluted plasma samples in 5 min [309]. They fabricated their sensing platform through RF magnetron sputtering of a semi-conducting ZnO layer (200 nm) onto gold surfaces, followed by antibody immobilisation and blocking using commercial SuperBlock (blocking buffer) used to hydrolyse unbound linker sites to avoid non-specific interaction. The ZnO film was used due to its large band gap (3.367 eV) and high excitation binding energy (60 eV) which both aid in increasing sensitivity. Additionally, the ZnO is non-toxic, has high adsorption, is chemically stable and possesses good electrical conductivity. EIS was used for the detection of specific binding between the antigen and antibody, achieving detection in a wide linear range of 0.01 pg mL^−1^ to 10 ng mL^−1^, with an LOD of 0.1 pg mL^−1^ for IL-6. They validated their results in pooled human blood plasma achieving a clinically accepted standard and an %RSD of ~ 10%, measured across 12 identical sensors. Further validation was obtained through the measurement of 40 patient samples (20 septic, 20 control) achieving a Pearson’s r value ≥ 0.9. This system shows the sort of validation required to provide confidence to professionals working outside of the electrochemical field.

#### C-reactive protein (CRP)

**Fig. 9:**
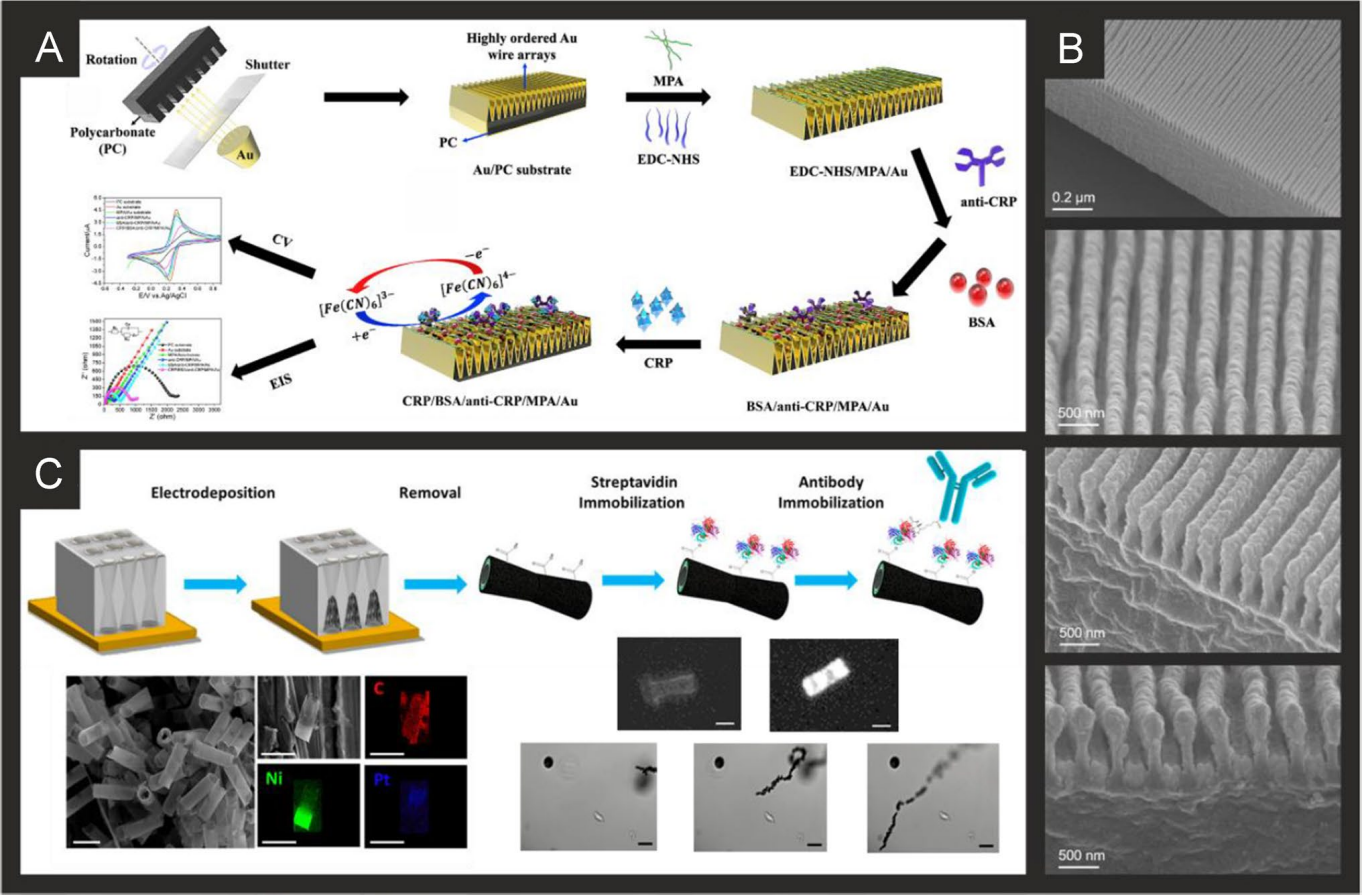
**A)** Schematic showing the fabrication of the gold wire sensor for CRP along with the detection strategy. Reproduced and adapted with permission from ref [323]. Copyright Elsevier 2019. **B)** Scanning electron microscopy images obtained at various magnifications of the Au/PC substrate used for the gold wire CRP sensor. Reproduced and adapted with permission from ref [323]. Copyright Elsevier 2019. **C)** Schematic of the preparation of rGO/Ni/PtNPs micromotors and their functionalisation with anti-CRP capture antibodies alongside SEM and EDX analysis (left), fluorescence microscopy images of the micromotors with and without streptavidin (right middle) and time-lapse images of the movement of the micromotors (right bottom). Reproduced and adapted with permission from ref [335]. Copyright American Chemical Society 2020

From inspection of Table 4, there is a wide variety of reported CRP electrochemical sensing platforms producing significantly different operational ranges due to the high concentrations of CRP present, see Table 1. As such, most reports in literature work through identifying the optimal detection ranges for their sensing platform and then diluting the samples for analysis by the appropriate factors. One such example is reported by Vilian and co-workers [323], who utilise a 100-fold dilution in the human serum samples for analysis. They report a simple immunosensor based on the formation of a 3-mercaptopropionic acid (MPA) SAM, followed by EDC/NHS coupling to the CRP antibody (Fig. 9A). The proposed sensor produced a linear range of 5 to 220 fg mL^−1^ with a low LOD of 2.25 fg mL^−1^, which is attributed to the gold nanowires grown on a polycarbonate surface. This gold surface was prepared through nanoimprint lithography using a customised electron beam evaporator, producing an Au film of 20 nm thickness (Fig. 9B). Detection of CRP was achieved through SWV of the [Fe(CN)_6_]^3−/4−^ redox couple, which exhibited a reduction in peak current (“signal off”) on increasing amounts of CRP. For real sample analysis, human blood serum was diluted 100-fold in buffer solution and subsequently spiked with varying amounts of CRP, with a LOD of 4.5 fg mL^−1^ achieved in this medium. Additionally, CRP detection was achieved in human saliva solutions using EIS, through a tenfold dilution in PBS (0.1 M), producing a LOD of 4 fg mL^−1^.

Molinero-Fernández and co-workers have described micromotor sandwich based immunoassays for the detection of CRP in preterm infant plasma [333, 335]. Micro (or nano) motors convert an external stimulus into autonomous propulsion and when used in a biosensing application this allows them to travel around a sample scavenging for the target analyte. They demonstrated the micromotors can be formed using rGO, MWCNT or carbon black (CB), with the rGO based systems producing the most efficient and reproducible functionalisation [333]. The formation of the micromachines uses a combination of rGO, Ni and PtNPs, shown in Fig. 9C. In this way, the rGO acts as functionalisation points for the CRP antibodies, the Ni layer allows for the magnetic guidance and the PtNPs provide an inner catalytic layer. Using these systems in conjunction with a microfluidic set-up and amperometric detection they were able to detect CRP between 1 and 100 µg mL^−1^ with a LOD of 0.54 µg mL^−1^. They used this to analyse CRP levels in preterm infant clinical samples with suspected sepsis, achieving readings using less than 10 µL sample volume in only 8 min [335]. These low sample volumes and quick turn-around time indicate the possibility of translation of this technology into clinical care.

An alternative methodology was presented by Szot-Karpińska et al. [327], who utilised bacteriophages as their recognition element. They immobilised these negatively charged bacteriophages onto a GCE surface in a layer-by-layer fashion with positively charged carbon nanofibers through electrostatic interactions. They compared systems with and without carbon nanofibers, showing that the biosensor using the CRP binding bacteriophage in conjunction with the nanofibers produced the best response, achieving a linear range of 4 to 40 µg mL^−1^ using three layers of the modification. They propose the use of phages as an artificial alternative to the traditional use of antibodies. We note that there are not many published works for the use of MIPs as another option for the replacement of antibodies with a more stable synthetic receptor. It is suggested this could be an area of research that is explored for CRP as the detection levels do not require the same sensitivities as many other biomarkers highlighted in this review.

Lastly, Cheng and co-workers have explored utilising the synergistic effect of AuNPs and melamine signal amplification through the use of a ferrocene modified small molecular peptide (Fc-ECG) as the bio-recognition element [339]. AuNPs were deposited onto the surface for carbon based SPEs via electrochemical deposition from a aqueous solution containing a gold salt, followed by the dropwise addition of melamine and then formation of Au–S bonds between the NPs and the Fc-ECG. The sensor worked through the free thiol group on the Fc-ECG binding with CRP to form larger complexes and inhibiting electron transfer from the ferrocene tag which produces a “signal off” sensor. Using DPV a linear range of 1 to 550 µg mL^−1^ was obtained with a LOD or 0.3 µg mL^−1^, achieving an %RSD of 4% in serum samples with good stability over 5 days post-production. For commercial uptake into clinical settings however a sensor lifetime longer than this would be required to reduce costs and wastage of tests.

#### Tumor necrosis factor α (TNF-α)

**Fig. 10:**
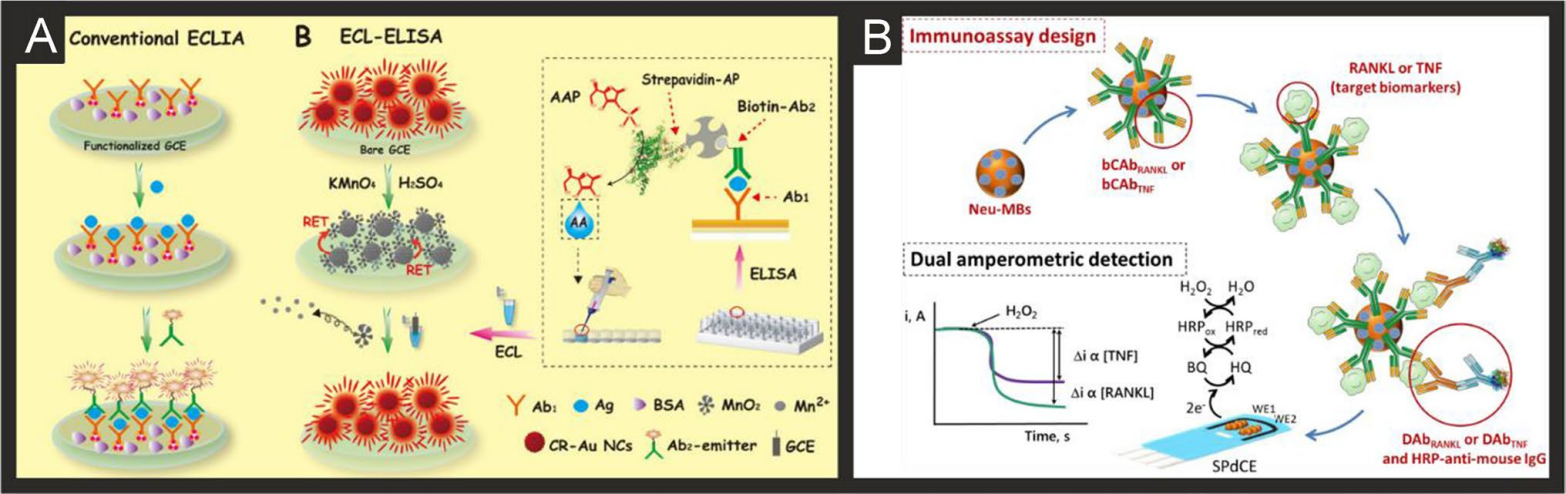
**A)** Schematic showing the bases of a conventional ECL-ELISA protocol and the ECL-ELISA protocol proposed by Peng and co-workers. Reproduced and adapted with permission from ref [356]. Copyright American Chemical Society 2019. **B)** Schematic showing the development of an MBs-based immune-platform for the dual amperometric detection of RANKL and TNF at dual SPCEs. Reproduced and adapted with permission from ref [358]. Copyright Elsevier 2020

As seen for the inflammatory markers above, on inspection of Table 4, there are a number of significant papers for the detection of TNF-α due to its association with a wide range of conditions. Peng and co-workers [356] have reported a versatile ECL based sensing platform utilising a GCE modified with gold nanoclusters (AuNC) and MnO_2_ (Fig. 10A). The AuNC were synthesized by dissolving a gold salt into sodium hydroxide with the reducing agent N-acetyl-L-cysteine. The mixture was incubated at 37 °C for 2.5 h, obtaining a colourless solution. The solution after synthesis was subject to dialysis for more than 24 h to remove all small-molecular impurity. MnO_2_ was introduced by modifying a GCE with the AuNC and electrodepositing MnO_2_ from immersing the electrode into a KMnO_4_ acidic solution and holding the potential at − 0.2 V for 300 s by chronoamperometry. They use an ECL-resonance-energy-transfer (RET) strategy, whereby the AuNC is the ECL donor and the MnO_2_ acts as the ECL acceptor. As seen in Fig. 10A, an ELISA based protocol is performed separately using antibodies functionalised with streptavidin and alkaline phosphatase. After the capture followed by enzymatic reaction has occurred, the resultant solution was collected and the modified GCE then incubated for 4 min. This platform achieved a linear response to TNF-α of 0.06 to 31 pg mL^−1^ with a LOD of 36 fg mL^−1^, which corresponds to a reduction in two orders of magnitude compared to commercial ELISA kits. They attribute the excellent performance of their sensor to the independence between the ELISA and ECL parts of the system, separating the sensing interface from the complex practical samples, in addition to the dual-signal amplification provided by the ECL technology and enzyme catalysed signal amplification.

The biofouling of electrodes is a common problem in the development of biosensing platforms, an alternative method to the separation of protocols mentioned above is the incorporation of anti-biofouling layers on the electrode. Jiang et al. [343] report using mixed layers of phosphorylcholine (PPC) and phenyl butyric acid (PBA) for the development of a TNF-α sensor in whole blood samples on an ITO electrode. In this zwitterionic mixed layer the PPC is responsible for repelling the non-specific protein adsorption that plagues many electrochemical biosensors, whereas the PBA allows for the bioconjugation of antibodies to the electrode surface. The authors then use a sandwich assay (“signal on”) in which the secondary antibody is labelled with HRP for amperometric determination of the presence of the antigen. Using this protocol, they achieved a linear relationship between the current response and TNF-α concentration between 0.01 and 500 ng mL^−1^ with a lowest detected concentration of 10 pg mL^−1^. They validated their sensor in whole blood against a commercial ELISA kit showing variations between 2.6 and 11.7%.

Valverde and co-workers reported the dual detection of two emerging biomarkers related to breast cancer, of which TNF-α is one [358]. They utilise an SPCE with two working electrodes, dropping the appropriate solution on each electrode respectively, Fig. 10B. This system utilises a sandwich immunoassay where the capture antibodies are immobilised onto neutravidin-modified magnetic beads, and detection antibodies are labelled with HRP. The binding of the antibodies to the target is achieved separate from the electrode surface in a centrifuge tube. After incubation, the solutions were washed and resuspended in buffer solution before being placed on the working electrodes. Amperometric measurements were used to achieve a dynamic range of 9.9 to 1,000 pg mL^−1^ for TNF-α, with a LOD of 3 pg mL^−1^. Their results were validated against a commercial ELISA, showing favourable results. The authors also show that the sensing platform can be stored at 4 ºC for 20 days with no significant differences observed in the sensitivities and there is no significant interference from a wide range of possible competitors. Their results were further validated against commercial ELISAs in human serum samples showing excellent agreement.

## Considerations for future research and progression into clinical care

Electrochemical approaches to PoC measurement of CBs in the critical care setting are extremely attractive given their speed, sensitivity, economy of production and ease of multi-panel integration. However, it is important to note that assay cut-offs have not been clearly established in this diverse patient population, and measurements during dynamic critical illness may be problematic. CB interpretation may also vary depending on individual patient characteristics and underlying illness.

From the literature on the design of the electrochemical sensing platforms, it is apparent that emphasis needs to be placed on the analysis procedure and time, production costs and storage life to provide added confidence in the technology. Most reports provide good validation for their work through measurements in real samples (human serum and plasma) and against commercial ELISAs, highlighting the promise of these technologies. The majority of work still utilises antibodies as their recognition elements and further work is expected toward using synthetic or man-made recognition elements to improve the batch-to-batch reproducibility, chemical and thermal stabilities, whilst also reducing the associated ethical concerns.

Additionally, we suggest the increased development of multiplexed sensing platforms to further enhance the possibility of commercial uptake of these technologies. As discussed, there are many markers that, although not specific for cardiac diseases, can be utilised in conjunction with the gold standard markers to provide crucial information to clinicians. In addition to increasing the confidence in the reported results, this will help to increase interest in the technology from external sources leading to increased chances of further funding or commercialisation. Future research should be directed to emerging immuno-thrombotic markers shown to be important in the diagnosis of cardiac diseases such as D-dimer and P-selectin [362–364], where electrochemical methods are currently limited [365–370].

## Conclusions

In this review, we outline the importance of rapid testing for CBs in critically ill patients, explaining the urgent need for developments in rapid, portable and sensitive sensing platforms. We highlight current gold standards used within clinical care in addition to discussing emerging CBs and their potential use in future, data driven patient care. We provide the sources of these CBs, along with their clinical relevance and desired analytical ranges found throughout the literature as reference points for future research on these CBs. We summarise the literature reported on the development of electrochemical sensing platforms for these CBs, focussing on the last 5 years for the most popular CBs, such as cTn’s. Additionally, we explore in detail some of the interesting recent developments for each CB, highlighting how the platforms are produced, function and what key characteristics they possess. Finally, the review offers insights on where we see the field developing and what needs to happen to improve confidence in these platforms and increase the chances of commercialisation and uptake into critical care, particularly with respect to ensuring that the technology focuses on wider dynamic ranges for measurement in this unique cohort of patients.
